# Factors influencing the implementation of mental health recovery into services: a systematic mixed studies review

**DOI:** 10.1186/s13643-021-01646-0

**Published:** 2021-05-05

**Authors:** Myra Piat, Megan Wainwright, Eleni Sofouli, Brigitte Vachon, Tania Deslauriers, Cassandra Préfontaine, Francesca Frati

**Affiliations:** 1 Douglas Mental Health University Institute, 6875, boul. LaSalle, Montréal, Québec H4H 1R3 Canada; 2 McGill University, Québec, Canada; 3 Department of Anthropology, Durham University, Durham, Canada; 4 School of Rehabilitation, Université de Montréal, C.P. 6128, succursale Centre-ville, Montreal, Québec H3C 3J7 Canada; 5 School of Rehabilitation, Université de Montréal, 7077 avenue du Parc, Montreal, QC H3N 1X7 Canada; 6 Université du Québec à Trois-Rivières, 3351 Boulevard des Forges, Trois-Rivières, QC G8Z 4M3 Canada; 7 Schulich Library of Physical Sciences, Life Sciences, and Engineering, McGill University, 809, Sherbrooke W, Montreal, Québec H3A 0C9 Canada

**Keywords:** Systematic review, Mixed methods, Mental health recovery, Recovery-oriented services, Recovery innovations, Implementation science, Consolidated framework for implementation research (CFIR), Best-fit framework synthesis

## Abstract

**Background:**

Countries around the world have committed in policy to transforming their mental health services towards a recovery orientation. How has mental health recovery been implemented into services for adults, and what factors influence the implementation of recovery-oriented services?

**Methods:**

This systematic mixed studies review followed a convergent qualitative synthesis design and used the best-fit framework synthesis method. Librarians ran searches in Ovid- MEDLINE, Ovid-EMBASE, Ovid-PsycInfo, EBSCO-CINAHL Plus with Full Text, ProQuest Dissertations and Theses, Cochrane Library, and Scopus. Two reviewers independently screened studies for inclusion or exclusion using DistillerSR. Qualitative, quantitative, and mixed methods peer-reviewed studies published since 1998 were included if they reported a new effort to transform adult mental health services towards a recovery orientation, and reported findings related to implementation experience, process, or factors. Data was extracted in NVivo12 to the 38 constructs of the Consolidated Framework for Implementation Research (CFIR). The synthesis included a within-case and a cross-case thematic analysis of data coded to each CFIR construct. Cases were types of recovery-oriented innovations.

**Results:**

Seventy studies met our inclusion criteria. These were grouped into seven types of recovery-oriented innovations (cases) for within-case and cross-case synthesis. Themes illustrating common implementation factors across innovations are presented by CFIR domain: Intervention Characteristics (flexibility, relationship building, lived experience); Inner Setting (traditional biomedical vs. recovery-oriented approach, the importance of organizational and policy commitment to recovery-transformation, staff turnover, lack of resources to support personal recovery goals, information gaps about new roles and procedures, interpersonal relationships), Characteristics of Individuals (variability in knowledge about recovery, characteristics of recovery-oriented service providers); Process (the importance of planning, early and continuous engagement with stakeholders). Very little data from included studies was extracted to the outer setting domain, and therefore, we present only some initial observations and note that further research on outer setting implementation factors is needed.

**Conclusion:**

The CFIR required some adaptation for use as an implementation framework in this review. The common implementation factors presented are an important starting point for stakeholders to consider when implementing recovery-oriented services.

**Supplementary Information:**

The online version contains supplementary material available at 10.1186/s13643-021-01646-0.

## Background

Mental health recovery is increasingly the focus of mental health policy, guidelines, and action plans worldwide. Recovery arose from the consumer survivor movement in the late 1980s as mental health service users began publishing on their own recovery experiences [1]. Personal recovery is not to be confounded with clinical recovery, the latter concept referring to measurable disease-focused outcomes such as a reduction in symptoms or hospital days. Personal recovery in contrast is defined as “a way of living a satisfying, hopeful, and contributing life even with limitations caused by illness” (p.527) [2]. Despite widespread adoption of the personal recovery concept, the challenge has been to operationalize the principles of personal recovery into services [3, 4], so that responsibility for recovery becomes a shared responsibility. While traditional mental health services focus on professional control, patient dependency, self-stigma, and hopelessness, the focus of recovery-oriented services is on client empowerment, choice, collaborative professional/client relationships, and community integration. In promoting a life beyond services, recovery also meets a key ethical obligation to honour the personhood and citizenship of people with mental illness.

Research on recovery has proliferated over the past two decades with studies on personal recovery [5–11], recovery-oriented services [12–19], and provider competencies [20–24]. Conceptual frameworks have been produced [25–30] and standardized measures have been developed [31–33]. Research has linked recovery to existing theories, e.g. empowerment theory [34, 35], the strengths model [36], capabilities theory [37–39], positive psychology [40–42], person-centered practice [43, 44], and co-production [45, 46]. Guidelines for recovery-oriented service provision are available [47–53].

To date, systematic reviews in mental health recovery have focused on conceptualizing personal recovery [25, 29, 54–60], measurement instruments [28, 61, 62], conceptualizing recovery-oriented practice [63], and intervention effectiveness [64–66]. One review described what influences the implementation of peer support work specifically [67]. However, no known systematic review, to date, has been published on how recovery has been implemented into services from an implementation science perspective. To address this knowledge gap, it was deemed appropriate to employ a systematic mixed studies review to ensure that we captured the breadth of evidence across research designs. This review seeks to address the question: How has mental health recovery been implemented into services for adults, and what factors influence the implementation of recovery-oriented services?

## Methods

### Synthesis design

This systematic mixed studies review follows a convergent qualitative synthesis design [68]. Based on Hong et al.’s classification of convergent synthesis design sub-types, ours can be described as “data-based”, meaning that findings from qualitative, quantitative, and mixed methods studies were extracted concurrently, analysed using the same method, and the review findings are presented together (p.7) [69]. We applied the best-fit framework synthesis method [70]. We chose the Consolidated Framework for Implementation Research (CFIR) [71] as the best-fit framework for this synthesis based on it being a germinal compilation of factors known to influence implementation and our aim being to systematically synthesize the factors known to influence the implementation of recovery-oriented services. The CFIR framework includes 38 constructs grouped into five domains: intervention characteristics, outer setting (outside or beyond the organization), inner setting (within the organization), characteristics of individuals, and process [71]. We used a hybrid deductive-inductive approach [68] that is consistent with the best-fit framework synthesis method [72]. The CFIR framework was used for data extraction by deductively coding findings from primary studies to the 38 constructs. Data within each CFIR construct was inductively coded thematically. Thematic synthesis methods are a common approach to mixed studies convergent qualitative synthesis design [68]. Currently, no published reporting guideline exists for systematic mixed studies reviews. We were guided by relevant elements of both the PRISMA [73] and ENTREQ [74] reporting guidelines (see Additional file 1). A comparison of the published protocol [75] and this review can be found in Additional file 2. A core team of three reviewers (MP, MW, ES) worked closely together on the review and kept a process log to document over 90 review meetings between December 2017 and the date of submission.

### Searches

A health sciences librarian conducted comprehensive searches in the following databases: Ovid- MEDLINE, Ovid-EMBASE, Ovid-PsycInfo, EBSCO-CINAHL Plus with Full Text, ProQuest Dissertations and Theses, Cochrane Library, and Scopus from January 1, 1998, to December 20, 2016, using a combination of keywords and MeSH terms. 1998 was chosen as the start date because it was in 1998 that recovery was first defined in an international policy document [76]. The search strategy was peer reviewed by another health sciences librarian using the Peer Review of Electronic Search Strategies checklist [77]. A third librarian (FF) updated the searches on July 25, 2018. No functional limits other than the start date were applied.

The search strategy was developed for MEDLINE (see Additional file 3), and a modification of this strategy was used to search the other databases. Two librarians executed all final searches (initial and updated (FF)), exported the results into EndNote and removed duplicates from the search results. A PRISMA flow chart was used to track the number of studies at each stage of the review. The table of contents of *Implementation Science, Psychiatric Services, Psychiatric Rehabilitation, Journal of Mental Health, Administration and Policy in Mental Health and Mental Health Services* were searched from January 2012 to March 2017. These journals were selected for having published several studies of interest to our review question. Eight researchers/experts in recovery and system transformation, from different countries, were contacted in July 2017 and asked to share any known empirical studies on the implementation of recovery into services published in the past 2 years or in press. No additional studies were identified through these means.

### Study inclusion and exclusion criteria

We included peer-reviewed studies that reported on the implementation process, factors, and experience when implementing new efforts to transform services for adults with serious mental illness towards a recovery-orientation. All study inclusion and exclusion criteria can be found in Table 1. All studies were independently screened over two stages for inclusion by two of three reviewers (MP, MW, ES) using DistillerSR software [78] (stage one: title and abstract. stage 2: full-text screening). Disagreements were resolved in meetings including a third reviewer.

**Table 1:** Inclusion and exclusion criteria

Inclusion criteria	
● Published peer-reviewed studies (qualitative/quantitative/mixed methods) investigating the implementation of recovery into adult mental health services for people with serious mental illness (e.g. schizophrenia, bipolar disorder, major depression) from the perspectives of staff, decision-makers, clients, and carers.	
● Studies reporting a new effort (within the organization or system) to transform services towards recovery-orientation and that is recovery-oriented in line with the definition of personal recovery by Anthony (1993) [2] (not clinical recovery).	
● Studies that include a description of the methodology for data collection/analysis in the abstract and full text.	
● Studies that report findings related to implementation experience, process, or factors.	
● Studies from any country and in any language.	
● Studies published from 1998 onwards.	
Exclusion criteria	
● Studies that describe interventions aimed at enhancing clinical recovery rather than personal recovery.	
● Studies on illness management and recovery (IMR), assertive community treatment (ACT), clubhouses, or psychosocial rehabilitation as these predate or do not emerge from the recovery movement and therefore were not considered “new efforts” (including more recent modifications of these—e.g. f-ACT).	
● Studies about employment or vocational services and personal budgets (though these are recovery-oriented they represent parallel literatures worthy of separate reviews).	
● Studies that describe innovations targeting the use of restraints and/or seclusion or studies whose primary outcome of interest was restraint and/or seclusion rates.	
● Studies reporting findings only about personal mental health recovery outcomes.	
● Studies solely about recovery in the context of addiction (substance abuse, gambling).	
● Reviews or systematic reviews, grey literature (e.g. reports, theses, dissertations, conference abstracts, editorials, letters), or conceptual papers.	
● Studies where the population of interest or service offered was specific to minors, youth, or young adults, including first-episode psychosis.	
● Studies that were about recovery in the context of natural disaster (e.g. earthquake, flood), physical health problems (e.g. stroke or cancer), eating disorders, mild depression, agoraphobia, postpartum depression, or domestic violence.	
● Studies about implementing education around recovery into undergraduate or postgraduate curricula (e.g. nursing, medicine, social work, occupational therapy).	
● Intervention effectiveness studies, implementation strategy effectiveness studies, and cost studies that do not report findings about implementation experience, factors, or process.	
● Author reflections on implementation process without evidence of a methodology.	
● Pre-implementation studies (change not yet implemented).	

### Study quality assessment

The Mixed Methods Appraisal Tool (MMAT) [79] was used to critically appraise all included studies. MMAT is a validated tool for appraisal of all study designs including mixed methods studies [80]. Studies were not excluded based on critical appraisal. Two reviewers independently appraised each study, using the MMAT template [79] and compared appraisals to arrive at a consensus (BV, TD, CP).^1^ Sensitivity analyses answer the question “are the findings robust to the decisions made in the process of obtaining them?” [81]. In this case, we conducted a sensitivity analysis to determine whether our decision not to exclude studies based on quality shaped the findings (e.g. are some findings based solely on lower quality evidence?). One reviewer (MW) applied Houghton et al.’s approach [82] to sensitivity analysis using matrix coding queries in NVivo 12 to visualize the number of studies by MMAT score categories supporting each theme presented in the findings section. The data coded to each theme were plotted against the MMAT score categories in the query. We scored the MMAT by counting “yes” responses and using five as the common denominator since both qualitative and quantitative studies are appraised based on five questions. Mixed methods studies are appraised on 15 questions so scores out of 15 were converted to scores out of five. This led to some scores with decimal points (e.g. 3.33). To simplify we assigned each study to one of four score categories: 0, 1–2, 3–4, and 5. Dividing studies into categories based on the number of critical appraisal criteria met is consistent with other studies that have applied sensitivity analysis to qualitative synthesis findings [70, 82, 83].

### Data extraction strategy

Descriptive data such as country, setting, objective, recruitment, data collection methods, theoretical framework, analysis, sample, and characteristics of participants were extracted to a form created in DistillerSR. Given that we used a qualitative approach to convergent synthesis [68] we approached the extraction of study findings in the following way: in qualitative studies, or the qualitative branch of mixed-methods studies, we extracted findings (quotes and authors’ descriptions) from results and discussion sections. In quantitative studies, or the quantitative branch of a mixed-methods study, we extracted the study authors’ own narrative descriptions and summaries of their quantitative results. Extraction of study findings to the CFIR was done in NVivo12 [84]. The data extraction template consisting of the five domains and 38 constructs of the CFIR [71], and their definitions, was pilot tested on five articles. Modifications were made including adding “authors and/or research participants descriptions of…” before each definition, adding an additional construct under a process called engaging with stakeholders, and adding an “additional information” code to each domain so that data that did not fit any of the constructs could be coded there and included in the thematic analysis (see Additional file 4 for data extraction framework used, including definitions).

In NVivo12, we created codes and sub-codes for each domain and construct and sub-construct and included the definitions in the “description” field of each code for easy access during extraction. PDFs of included articles were imported, a case node was created for each article, and all were coded to the case node “included studies”. A case classification sheet was created with descriptive information about the studies (country, perspectives). This process enabled the use of data exploration features in NVivo12, such as framework matrices and matrix coding queries. Data was extracted by coding sections (e.g. a sentence, quote or paragraph) of the PDF to corresponding CFIR constructs. If a section of data illustrated more than one construct, it was coded to each. Factual information provided by the authors that related to CFIR constructs (this usually appeared in background and methods sections) were extracted to a second coding tree but not used in this review.

One reviewer (MW) carried out data extraction. Five studies were co-extracted by a second review (MP, ES) for quality control. Three reviewers (MP, MW, ES) met weekly over this stage to discuss extraction and interpretations of CFIR constructs. Questions about interpreting distinctions between domains and constructs were clarified in a meeting with authors of the CFIR.

### Data synthesis and presentation

Due to the wide variety of innovations implemented with the aim of transforming services, three reviewers (MP, MW, ES) worked to conceptually group the 70 included studies into similar types of innovations, as a precursor to analysis. Each innovation group became a “case” and we created case nodes for each innovation group in NVivo12. This facilitated analysis and synthesis conceptually by enabling the reviewers to embed themselves in the extracted data case by case. Table 2 shows each innovation group (case) and the number of studies in each (it also highlights studies that were coded to more than one innovation group (case)—e.g. a study of peer workers doing personal recovery planning. An additional case node was created called “perspectives on implementation of recovery-oriented services in general” for those studies with a broader focus and without enough description of the intervention to enable grouping. These, alongside innovation categories with only one supporting study, were not included in the within-case and cross-case synthesis. In total, 55 studies representing seven innovation groups (cases) were included in a within-case and cross-case analysis (thematic coding within CFIR constructs) and synthesis (writing summarized review findings). Analysis and synthesis in NVivo12 was undertaken by one reviewer (MW) who met weekly with two reviewers to discuss emergent findings and co-interpret data (MP, ES). The following details the steps and procedures of the within-case and cross-case analysis and synthesis.

**Table 2:** Innovation groups, definitions, and corresponding studies

	Innovation group (cases)	Definition	Studies*
1	E-innovations	Online innovations such as websites and smartphone apps.	[85–90]
2	Family-focused innovations	Innovations specifically aimed at mental health service users who are parents.	[91–93]
3	Peer workers	Innovations centred on the employment of people with lived experience of mental health problems.	[94–104], [105–109], [110], [111–115]
4	Personal recovery planning	New approaches to writing plans within service provider–service user encounters.	[109, 116–121], [115, 122], [123, 124]
5	Recovery colleges	Education programs offering courses to service users and service providers on recovery and other topics in mental health.	[125–129]
6	Service navigation and coordination	Innovations aimed at wraparound care, care coordination, and client access to services across health and social services.	[104, 130, 131], [110, 132], [133, 134]
7	Staff training	Training programs for staff in mental health recovery.	[18, 135–139]
8	Architecture	Not included in synthesis. See Additional file 7 for details.	[140–144]
9	Community connections		
10	Consumer-led advisory councils		
11	Personal budgets		
12	Sport		
**Other**			
Perspectives on implementing recovery-oriented services in general		Not included in synthesis. See Additional file 7 for details.	[115, 145–153]

^*^The following studies appear under more than one innovation group because the innovation crosses two categories and findings related to each are reported [104, 109, 110, 155]. For Smith-Merry et al. [155], only the data reported about peer workers and wellness recovery action planning were included in Synthesis Part 2

#### Within-case analysis

The data extracted to each CFIR construct for each separate innovation group (case) were thematically coded. In Nvivo12, we generated a framework matrix table for each innovation group into which we wrote-up summarized review findings under each theme while easily viewing the extracted data coded to the theme. Summarized review findings were saved to seven documents, one per innovation group (case). The principal investigator (MP) read and commented on all within-case review findings and read all the data underlying each finding to verify that the findings were adequate representations of the extracted data.

#### Cross-case analysis

The seven documents containing the within-case review findings for each innovation group were imported to NVivo12 as the data set for the cross-case analysis and synthesis. Each document was coded to its corresponding innovation case node and each finding coded to its corresponding CFIR domain and supporting studies. This ensured traceability within NVivo12 between the summarized findings and the underlining data extracted. For cross-case analysis within-case findings from all innovation groups were thematically coded by CFIR domain, starting with intervention characteristics and ending with process. First, summarized findings were coded to the categories “common” (findings that emerged across innovations) and “specific” (findings that related specifically to one innovation). This review focuses on factors common across innovations (cases) that influenced implementation.^2^ The within-case findings categorized as “common” were grouped into cross-case themes. The list of emergent themes was divided into primary and secondary themes. Primary themes were those that occurred across the most innovations (cases) and were best supported by the data. An NVivo12 framework matrix was created for each domain to assist with merging innovation-specific findings into a single narrative illustrating a cross-case theme. Theme names were refined in the process. These represent the final results of this synthesis (Table 3). To reduce the length of the manuscript, we do not report on two primary themes within inner setting (financial issues and staff time) since we believe these factors are widely known and reported on in the implementation science literature. The sustainability of funding and staff perceptions of the time they have for implementation are also fundamental factors to consider when implementing new innovations. We briefly summarize outer setting rather than present findings because this was the least well-supported domain and requires further research.

**Table 3:** CFIR domains, synthesis themes, and corresponding CFIR constructs data were extracted to

CFIR domains	Name of themes from the synthesis	CFIR construct(s) where data underlying this theme were coded to
Intervention characteristics	● Flexibility	● Design quality and packaging ● Relative advantage ● Adaptability
	● Relationship building	● Design quality and packaging ● Complexity
	● Lived experience	● Design quality and packaging ● Relative advantage ● Source of the Intervention
Inner setting	● Traditional biomedical vs. recovery-oriented approach	● Culture ● Learning climate ● Compatibility ● Relative priority
	● The importance of organizational and policy commitment to recovery-transformation	● Compatibility ● Leadership commitment ● Tension for change
	● Staff turnover	● Structural characteristics
	● Lack of resources to support personal recovery goals	● Available resources
	● Information gaps about new roles and procedures	● Access to knowledge and information
	● Interpersonal relationships	● New construct: Relationships
Characteristics of individuals	● Variability in knowledge about recovery	● Knowledge and beliefs ● Self-efficacy ● Individual stage of change
	● Characteristics of recovery-oriented service providers	● Other personal attributes
Process	● The importance of planning	● Planning
	● Early and continuous engagement with stakeholders	● Engage: (new construct) engaging with stakeholder ● Reflecting and evaluating ● Formally-appointed internal implementation leader

*CFIR* Consolidated Framework for Implementation Research

## Results

### Included studies

**Fig. 1:**
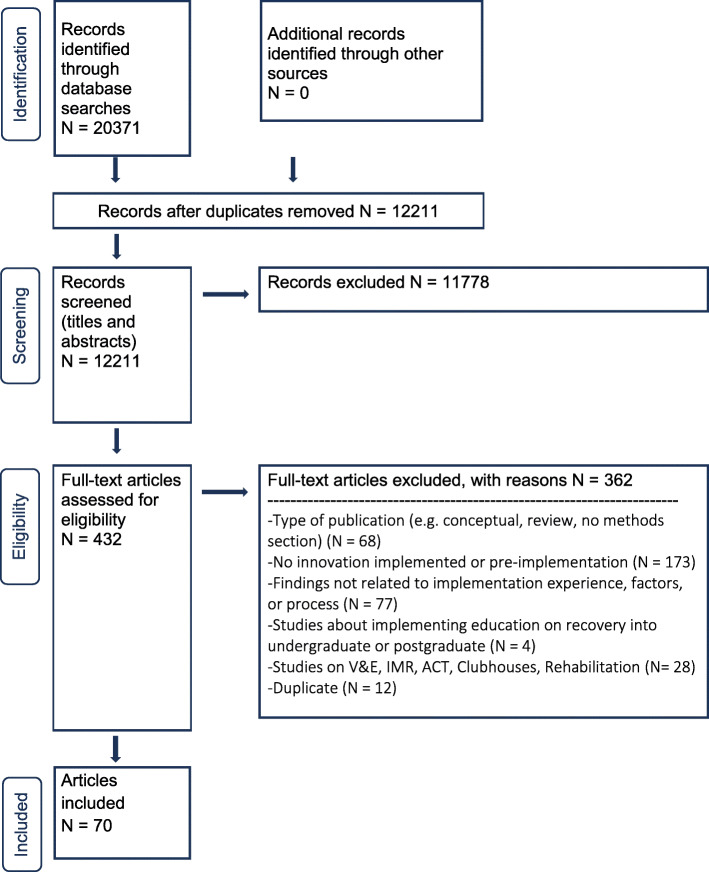
PRISMA flow chart. V&E, vocation and employment; IMR, illness management and recovery; ACT, assertive community treatment

Figure 1 is the PRISMA flow chart representing the stages of study selection for this review. In total 70 studies (publications) met our inclusion criteria and were included in this review (see reference list in Additional file 5). Eleven studies originated from four research projects [18, 85, 88, 97, 103, 124, 145–148, 151] but each publication was treated as an individual study. We reflected on whether this decision impacted our findings by using the Query function in NVivo12 to see how many publications contributing to a theme were from the same study. Only four of the 13 themes presented in the synthesis section below were contributed to by two publications from the same study. We therefore conclude that none of the themes are overrepresented by data emanating from a single project.

### Results of study quality assessment and sensitivity analysis

Of the 70 included studies, 55 were categorized as qualitative, six mixed methods, six quantitative descriptive, one quantitative non-RCT, one quantitative RCT, and one as both quantitative descriptive and qualitative for the purposes of the MMAT assessment. When only part of the findings were relevant to this review, the study was categorized according to the methods used to produce these findings only (hence the categorizations may not match the design of the whole study). For example, if a study collected qualitative and quantitative data but only the qualitative component related to our review question, we categorized the study as qualitative for MMAT. We categorized as mixed methods studies that self-described as such or that collected, analysed, and integrated both quantitative and qualitative data. Studies that did not demonstrate any integration and did not self-label as mixed methods were categorized as “quantitative and qualitative” and both sets of questions in MMAT were used. Overall, the majority of included studies were good quality studies, with 35 studies scoring 5, and 27 scoring as 3–4. There were just as many or more “Can’t Tell” responses compared to “No” suggesting that in some studies lower appraisals may reflect issues in reporting rather than actual quality. Resources were not available to contact authors to clarify “Can’t Tell” appraisals. Appraisals can be found in Additional file 6.

**Fig. 2:**
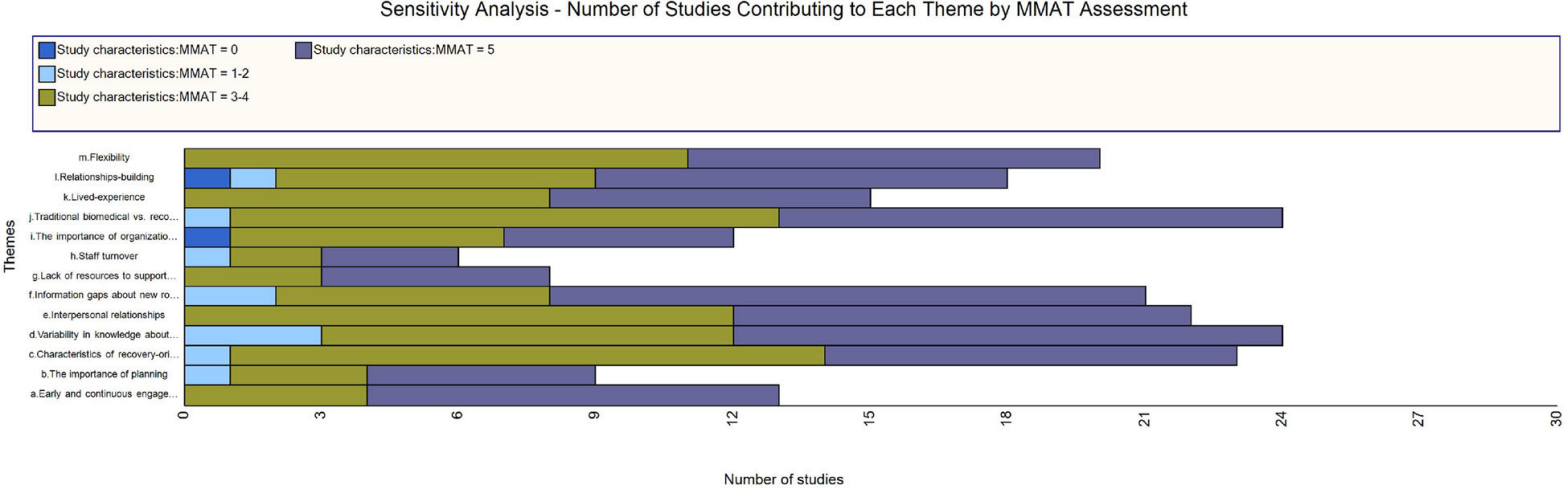
Results of the sensitivity analysis showing how many studies of each appraisal category contribute to each theme. MMAT, Mixed Methods Appraisal Tool

Figure 2 shows the results of a sensitivity analysis of the themes presented under each CFIR domain. We conclude that none of the themes are based solely on lower quality studies and that all themes are well supported by higher quality studies.

### Synthesis

Although we extracted data from and appraised all 70 studies, we chose not to include 15 studies in the within and cross-case synthesis either because they were in innovation groups containing only one study, or they were studies exploring perspective on implementing recovery-oriented services in general (see Table 2). For the 55 studies categorized into one of the seven innovation groups, Table 3 lists by CFIR domain the themes representing common implementation factors synthesized from across these studies. The table also shows the corresponding CFIR constructs to which the data underlying the theme were coded to at the data extraction phase. Illustrative quotes from contributing studies for each theme can be found in Additional file 8. In the following, we describe the general pattern observed across multiple innovations and provide innovation-specific examples in tables (one per CFIR domain).

#### Intervention characteristics

##### Flexibility

Across innovations, flexibility was highlighted as an important intervention characteristic that enhanced adaptability and was sometimes seen to provide a relative advantage over traditional services [85, 88, 89, 93, 101, 104, 109, 110, 114, 115, 117, 125–127, 129–131, 133, 134]. For example, having flexible program content that service providers and service users could pick and choose from and tailor to their own, and their clients’ needs were valued [85, 88, 89, 93, 115, 117, 125, 127, 129]. Flexible role definition for service providers (including peer workers) delivering recovery-oriented services was also valued as it enabled tailoring services to service user and community needs [101, 104, 109, 110, 131]. Service providers could more easily adapt to the needs of service users if the elements surrounding the innovation’s delivery, such as location, mode, timing, frequency, structure, or length were flexible [93, 101, 109, 110, 125, 126, 134]. Flexible funding was another characteristic of some interventions that service providers found helpful in adapting their support to service users’ needs [114, 130, 133]. Table 4 provides examples of flexibility for each innovation group.

**Table 4:** Intervention characteristics: themes and examples from each innovation group

Innovation group	Theme^**a**^	Example
**E-innovations**	Flexibility	Some service users appreciated the flexibility the online portal offered compared to in-person consultations because these were available 24 hours a day, and they offered a diversity of modules and content that could be adapted to each clientʼs needs and interests [85, 88, 89].
	Lived experience	Incorporating videos of people with lived experience of mental illness was valued by some, but not all users [89, 90].
**Family-focused innovations**	Flexibility	Flexible program content for group sessions enabled the facilitators to tailor the program to meet the unique needs and context of the particular group (for example based on the age of participantsʼ children) [93].
**Peer workers**	Flexibility	Flexibility in defining peer workers’ roles [104, 115] and in terms of the timing, structure, and frequency of sessions with service users [101, 109] helped peer workers adapt their services to service user and community needs.
	Relationship Building	Managing relationships with staff and service users can be a complex process due to peer workers having to shift identities from that of a service user to that of a service provider, while at the same time continuing to juggle these identities in their work [102, 105, 114]. Their role is to develop close trusting relationships with service users but managing boundaries and ending relationships can be emotionally complex [99, 104, 109, 114].
	Lived experience	Peer workers were thought to have an advantage compared to clinical staff because they enable greater control over choices rather than tell clients what to do [98, 104, 109], and were less controlling and intrusive and could be trusted because they did not have the power to take away service users’ rights [106]. Because of their lived experience they are more credible and trusted [104, 106, 109] and service users open-up more to them [103, 104, 106, 114].
**Personal recovery planning**	Flexibility	Workbooks and guides could help structure the process of recovery planning, but flexibility was important for the acceptability of the intervention among staff and clients (in terms of being optional, tailoring it to service users’ interests, including unstructured space (e.g. for drawing) and adapting to service usersʼ pace) [115, 117].
	Relationship building	Personal recovery planning involves close relationship building between service providers and service users that entailed a certain amount of complexity around managing the relationship, navigating boundaries, and dealing with a sense of loss when the relationship was required to end at the end of the intervention [109].
	Lived experience	Recovery planning interventions designed or co-designed by people with lived experience was seen as an important design feature [109, 115, 116].
**Recovery colleges**	Flexibility	Designing the college so that all students could easily join and sign-up for courses without need for referral or prerequisites was highly appreciated, as was being able to make oneʼs own choices of what to take, how much to participate in class, and dropping a course without being penalized [125, 127, 129].
	Relationship building	Practitioner tutors can experience some challenges related to negotiating their dual role of colleague and clinician if the peer co-tutor is also their client and becomes unwell while working together [126].
	Lived experience	Including people with lived -experience as peer tutors delivering recovery college courses was valued because of their insight into what people are going through, because students could identify with them, and because their stories of recovery inspired hope and optimism among staff and service user students [125, 128, 129].
**Service navigation and coordination**	Flexibility	In comparison to traditional case management, service navigation and coordination initiatives appeared to have fewer boundaries—for example service providers could do whatever it took to support recovery, and could meet clients in the community rather than in an office [110, 134].
	Relationship building	Relationships are formed between service navigators/coordinators and service users and there was concern on both sides about managing program exiting, transitions to other programs, and scaling back frequency of contacts [133].
**Staff training**	Lived experience	Including people with lived experienced, for their real-life inspirational examples of recovery, and the sense of equality they brought to sessions, increased comfort, encouraged openness, and challenged prior identities as professional or service user [135, 137].

^a^If the studies in the innovation group did not contribute data to a theme, that theme is not listed under the innovation group and no example is provided

##### Relationship building

Relationship building is a key element of innovations aimed at transforming services towards a recovery orientation. A common characteristic of recovery-oriented innovations is the appointment of a service provider who takes on a role predicated upon building trusting relationships with service users (e.g. facilitator) [85, 87, 89, 90, 93, 109, 116, 118, 120, 122]. However, relationship building is also an element of these innovations’ complexity from the point of view of service providers, both in terms of managing relationships with co-workers and service users, and ending relationships with service users [99, 102, 104, 105, 107, 114, 118, 126, 133]. Table 4 provides examples for each innovation group.

##### Lived experience

Across innovations, the inclusion of people with lived experience of mental health challenges was a valued aspect of designing and packaging recovery-oriented innovations [89, 90, 98, 103, 104, 106, 109, 114–116, 125, 128, 129, 135, 137]. When the source of the intervention was a person or group of people with lived experience, this was viewed positively [109, 115, 116]. Those with lived experience were perceived to have a relative advantage over other staff when it came to working in a recovery-oriented way [98, 103, 104, 106, 109, 114]. Table 4 provides examples for each innovation group.

#### Outer setting

Very little data was extracted to outer setting. The question of how well organizations knew the needs and resources of their clients (as it is framed in CFIR) was rarely directly studied or reported on in the findings. We extracted data to patient needs and resources that spoke generally about service users’ needs but because we judged that this data did not capture the meaning implied by the CFIR construct we do not present it here. External policies were a topic covered descriptively in the background sections of articles rather than directly studied and reported on in findings, and so relatively little data on the topic contributed to this review. One finding worth mention is that external policies of funders and governments regarding billing for services (in the USA especially) caused difficulties for peer worker and service navigation and coordination innovations when these new roles and services did not easily fit existing funding structures [96, 134].

#### Inner setting

##### Traditional biomedical vs. recovery-oriented approach

Data extracted and synthesized to three CFIR constructs (compatibility, culture, learning climate), spoke to the overall theme of the challenge with implementing recovery into services that espouse the medical model. Across innovations stakeholders perceived there to be compatibility issues between traditional organizational culture (described in terms of hierarchies, unequal power relations, paternalism, and punishment) and recovery-oriented innovations [94, 98, 99, 103, 104, 108, 111, 115, 119, 122, 124, 131, 134–137]. An important aspect of traditional organizational or service culture is the way in which staff understand their roles and the priorities of their job. Traditional roles and priorities, such as dealing with acute episodes of mental illness, a focus on medication prescription, and managing risk, were not easily compatible with implementing recovery into services [18, 86, 92, 100, 108, 135]. Many peer workers described a culture of stigma towards mental illness in the organizations in which they worked (and the field of mental health in general) that affected both them and the clients they served [96, 99, 102, 108]. The learning climate for service users—that is, how safe services users feel to try new recovery-oriented services—was sometimes poor because traditional organizational culture from the point of view of some service users is one of distrust and fear due to past negative experiences (e.g. unpleasant or forced treatments) [119, 124, 132]. Specific examples by innovation group are in Table 5.

**Table 5:** Inner setting: themes and examples from each innovation group

Innovation group	Theme*	Example
**E-innovations**	The importance of organizational and policy commitment to recovery-transformation	An e-innovation was welcomed by leaders because they saw it as helping the organization progress towards their policy goals of measuring and increasing user involvement in care plans [85].
	Interpersonal relationships	Service users were excited to use the e-innovations but disappointed and frustrated when their providers did not participate in and support them as much as they expected them to. Some providers felt their clients’ expectations were difficult to fulfil [85, 88]. A positive learning climate was thought to be linked to good pre-existing working relationships between service users and service providers, particularly ones that were open and adaptable [85, 88].
**Family-focused innovations**	Information gaps about new roles and procedures	The need for establishing guidelines, protocols, and procedures to help staff implement family-focused innovations was highlighted [91, 92]. Nurses in the family rooms innovation were unsure if they should or should not stay with families during visits, and what their role was during visits, which left them feeling uncertain and having to navigate as best they could [92].
	Interpersonal relationships	The fact that the group members and the facilitator already knew each was thought to have helped establish the trusting relationships and cohesive group dynamic that were key to successful implementation [93].
**Peer workers**	Traditional biomedical vs. recovery-oriented approach	Peer workers often felt that other staff, primarily mental health professionals and doctors, valued their own knowledge (gained through formal degrees) more than peer workersʼ knowledge (gained through lived experience) [94, 104, 108, 115], with some describing feeling “blown-off” [108] and treated like a “kid”, an “idiot”, or a “moron” [99] in the workplace, and that any change in mood or any day off work was assumed to be related to their mental health problems [99, 108].
	The importance of organizational and policy commitment to recovery transformation	If there was a lack of compatibility between the peer worker philosophy and the existing paperwork, treatment plans, and requirements for stating goals and demonstrating progress that they were asked to use, peer workers could feel uncomfortable with, and critical of, the service they provided their clients [94, 99].
	Information gaps about new roles and procedures	Peer workers often lacked information about their roles and tasks [104, 108]. A commonly reported issue was the lack of training and information for non-peer staff about the peer worker role, recovery, and how to work with (or supervise) peers workers [94, 97, 100, 102, 108]. This could lead to the underutilization or misutilization of peer workers [97, 108], and role confusion and conflict [102, 108].
	Interpersonal relationships	Building good interpersonal relationships between peer workers and non-peer staff was important for increasing respect and acceptance of the peer worker role [98, 108], ensuring the peer workers' role and skills were fully utilized [105], and facilitating the transition of the peer worker from service user to service provider [106, 114]. Hiring peer workers from within an organization’s own client population came with certain challenges due to pre-existing relationships [99, 102].
**Personal recovery planning**	Traditional biomedical vs. recovery-oriented approach	Traditional mental health services espouse independent and distinct responsibilities whereas recovery planning requires cooperative and collaborative teamwork that shares responsibility among staff [119].
	The importance of organizational and policy commitment to recovery transformation	Personal recovery planning can risk becoming just another skill to acquire or just another care plan to complete in a formulaic and non-individualized way if wider organizational change does not occur [115, 119, 124].
	Staff turnover	Difficulty retaining staff and filling key positions meant that building a continued vision for recovery planning as part of wider organizational change was difficult [119].
	Lack of resources to support personal recovery goals	Service providers perceived there to be a lack of resources for supporting clients’ individually-determined goals in a hospital setting because there was limited programming available [119].
	Information gaps about new roles and procedures	Service users and service providers need access to clear information about the role of the service provider, the purpose of personal recovery planning and benefits for service users, and how the recovery plan will be communicated to others on the team and physically stored [119, 122].
	Interpersonal relationships	Positive relationships were characterized by respect and mutual esteem and negative ones as being told what to do and being patronized [109, 117, 122]. When staff were disinterested in recovery plans or had negative attitudes towards the training and additional paperwork needed, clients perceived this lack of buy-in and felt disappointed, concerned, or equally dismissive of aspects of recovery planning [119, 121, 122].
**Recovery colleges**	Information gaps about new roles and procedures	Guidance was needed for service provider students about how to manage boundaries in co-learning environments and whether they should or should not disclose their status as a member of staff to others [128].
	Interpersonal relationships	Achieving good rapport between practitioner and peer tutors paired-up to teach courses may be more difficult to achieve if the practitioner tutor is normally the peer tutorʼs service provider [126].
**Service navigation and coordination**	Traditional biomedical vs. recovery-oriented approach	Overcoming existing traditional work culture involved dispensing with hierarchical structures, competitiveness, and defensiveness that can silo or make invisible scarce community resources [129], working in a more intensive and individualized way with service users [132], and pre-empting challenges inherent to a historical separation between behavioural and physical health [132].
	Staff turnover	Turnover could cause unclear leadership and inefficiencies since what staff are required to do may keep changing as people in leadership roles change [130].
	Lack of resources to support personal recovery goals	Service navigation and coordination depends implicitly on the availability of external services to coordinate, but the lack of services to actually coordinate can threaten its purpose [130, 132–134].
	Information gaps about new roles and procedures	Lack of access to information and training around the new service navigation and coordination programs and the role of its staff (processes, referrals, expectations, goals, outcomes, funding, philosophy) was mentioned across studies and was associated with stress, concerns, confusion, difficulties with service navigation, and more difficult relationships with other service providers [110, 130, 131, 133, 134].
	Interpersonal relationships	Trusting, supportive and caring relationships seemed to be a central factor for service user satisfaction and positive change in service navigation and coordination innovations [130, 133].
**Staff training**	Traditional biomedical vs. recovery-oriented approach	Recovery training was occurring in an organizational culture characterized by hierarchies and unequal power relations (between different staff, and staff and service users) [133, 136], and one in which self-reflection was a rare occurrence [134].
	The importance of organizational and policy commitment to recovery-transformation	Staff supported the view that organizational culture (mission, policies, procedures, record-keeping, staffing) needed to change in order for implementation of a recovery training program to be successful [135].
	Staff turnover	In one study staff turnover was 21% during the training program [135], and in another study, 15% of staff in one site, and 37% in another site left their jobs during the training intervention [139].
	Lack of resources to support personal recovery goals	Outside of hospital settings, there may be a lack of resources to draw on to help service users meet their full potential [18], including community resources such as appropriate placements and accommodation [135].

*If the studies in the innovation group did not contribute data to a theme, that theme is not listed under the innovation group and no example is provided

##### The importance of organizational and policy commitment to recovery-transformation

The compatibility between the wider organizational commitment to recovery-oriented transformation and the recovery innovation being implemented was important for staff [85, 86, 115, 119, 124, 135], especially peer workers [94, 98, 99, 102, 107, 115]. Staff, including peer workers, expressed concern that if recovery-oriented innovations are implemented into a wider organizational setting that does not espouse the same recovery values, then the success of the innovation will be hampered [99, 115, 119, 124, 135]. Leadership and staff buy-in for an innovation is enhanced by a perception that the innovation is compatible with existing organizational and service goals [85, 86]. Specific examples by innovation group are in Table 5.

##### Staff turnover

Structural characteristics of organizations such as staff turnover and difficulty recruiting and retaining staff were mentioned as implementation challenges across innovation groups [18, 119, 130, 132, 135, 139]. If staff or managers change frequently, the climate for implementation may be compromised by increased workloads, staff stress, and changes to teams’ skill mix [18], and can lead to inefficiencies in building capacity and a continued vision for recovery in the organization [18, 119, 130, 135, 139]. Specific examples by innovation group are in Table 5.

##### Lack of resources to support personal recovery goals

An aspect of many recovery-oriented innovations is to support service users in their own personal recovery journeys by facilitating access to the resources and services they wish to make use of. A challenge which sometimes arose across different innovation groups was a lack of available resources beyond the innovation both within the organization and in the community for supporting personal recovery goals, such as relevant programming, services, placements, and accommodation [18, 119, 130, 132–135]. Specific examples by innovation group are in Table 5.

##### Information gaps about new roles and procedures

The need for additional guidance and training to help clarify roles and specific procedures within innovations was mentioned across studies. This need was mentioned not only for service providers delivering an innovation but also for those who supervised them, other service providers working alongside them, and the service users with whom they worked [91, 92, 95, 96, 98, 100, 104, 108, 110, 119, 122, 128, 130, 131, 133, 134]. When recovery innovations involve the implementation of new roles, lack of access to information about the new role can cause a number of difficulties in the workplace including added stress, confusion, difficult relationships, and work [104, 108, 110, 130, 131, 133, 134]. Service users, new staff, and existing staff also noted needs for greater access to information about new procedures, roles, or services on offer [91, 92, 94, 97, 100, 102, 108, 119, 122, 128]. Specific examples by innovation group are in Table 5.

##### Interpersonal relationships

As reported under intervention characteristics, relationship building is both a key design feature and a source of complexity for recovery-oriented innovations. Interpersonal relationships play out in the inner setting and can be helped or hindered by existing relationships. Since the CFIR framework does not have a construct related to relationships in the inner setting we developed an additional construct called “interpersonal relationships” from the data extracted to additional information. Building good interpersonal relationships between existing staff and newly hired service providers taking-up new roles is an important factor for innovation success [98, 105, 106, 108, 114, 131]. So are positive interpersonal relationships (described as trusting, respectful, mutual esteem, supportive, and caring) between staff providing recovery-oriented services and service users [109, 117, 122, 130, 133]. Managing expectations within the service provider-service user relationship was also an important element of positive interpersonal relationships and fostering a positive implementation climate [85, 88, 119, 121, 122]. Pre-existing interpersonal relationships between staff or staff and service users can at times facilitate [85, 88, 93] or pose certain challenges [99, 102, 126] to implementation. Specific examples by innovation group are in Table 5.

#### Characteristics of Individuals

##### Variability in knowledge about recovery

The issue of variability in understandings of the concept of recovery was mentioned across studies [18, 86, 92, 94, 99, 102, 109, 113, 117, 119, 121, 122, 129, 135–137, 139]. While a good understanding of recovery principles was specifically noted in some studies [109, 117, 122, 135], in others, some non-peer service providers still confounded personal and clinical recovery [92, 94, 99, 102], and expressed a belief that not all service users could participate in recovery-oriented services because they lacked some necessary quality or level of wellness [86, 129, 136, 137, 139], thus demonstrating a lack of familiarity with the facts, truths, and principles of a recovery-orientation. Examples by innovation group are in Table 6.

**Table 6:** Characteristics of Individuals: themes and examples from each innovation group

Innovation group	Theme^**a**^	Example
**E-innovations**	Variability in knowledge about recovery	Some doctors in an e-innovation study showed more interest in less-recovery-oriented aspects of the innovation, such as the tool’s capacity for clinical monitoring of sleep and symptoms [86].
**Family-focused innovations**	Variability in knowledge about recovery	In one study of a family-focused innovation, nurses tended to confound personal and clinical recovery (e.g. they referred to recovery as the clinical improvement of symptoms and a process of regaining physical and mental health to a point where the client could be discharged) [92].
**Peer workers**	Variability in knowledge about recovery	Some peer workers felt strongly that recovery and the roles of peer workers had been misunderstood and co-opted in the mental health system, that they were being asked to do tasks and roles that contradicted the recovery approach or that trivialized their role (being a clerk or a driver), and that some clinicians misused the term and confused clinical recovery with concepts of personal recovery [94, 99, 102].
	Characteristics of recovery-oriented service providers	Personal attributes of peer workers that facilitated or optimized their work and impact included: patience [99, 111], being warm and understanding [106, 109], dependable and trustworthy [106, 111], professional, a good communicator and listener, respectful (didnʼt dictate), empathetic, positive, and optimistic [111].
**Personal recovery planning**	Variability in knowledge about recovery	Staff and clients showed familiarity with the facts and truths about the recovery plan when they expressed understanding that it was both process and outcome [122], owned by clients [117] and personalized [109]. However, some staff made judgements about their service usersʼ goals, such as not being realistic or not meaningful [119] and some clients did not understand the underlying concept of mental health recovery and thought the plan was a once-off thing [121].
	Characteristics of recovery-oriented service providers	Positive experiences were related to finding facilitators supportive, respectful, encouraging, helpful, collaborative, and warm [109, 117, 124]. Negative experiences were related to perceiving facilitators as patronizing in their approach, not genuine in their compassion or formulaic and generic in their approach, or having done an inadequate job discussing recovery [109, 121, 122].
**Recovery colleges**	Variability in knowledge about recovery	Some service provider students in recovery colleges felt that service users needed to be well enough mentally to participate [129].
**Service navigation and coordination**	Variability in knowledge about recovery	Even when state officials are very clear on the distinction between dependency-producing case management and self-managed recovery, and providers excited by the new model and open to client empowerment, in practice the two can become blurred [134].
	Characteristics of recovery-oriented service providers	Success of service navigation and coordination innovations appeared closely tied to personal characteristics of staff, in particular the ability to develop strong individual connections, trust, and rapport with both clients and other services through a personal approach, addressing competitive or defensive responses, empowering themselves, being hardworking, and having the skills to navigate fragmented systems [130, 131, 133].
**Staff training**	Variability in knowledge about recovery	While the centrality of hope and recovery-oriented language was understood, some, despite training still thought of recovery as a linear journey with a start and end point, or as a type of care, or something they did for clients [135]. Some staff were concerned that many service users may not be at a level of recovery necessary to engage in a recovery training process [137].

^a^If the studies in the innovation group did not contribute data to a theme, that theme is not listed under the innovation group and no example is provided

##### Characteristics of recovery-oriented service providers

The experience of the innovations and their relative success were considered to be closely tied to the specific attributes of the service provider delivering the recovery-oriented service [99, 104, 109, 111, 117, 121, 122, 124, 130, 131, 133]. Positive experiences and implementation success were related to positive personal attributes including being respectful, encouraging, helpful, collaborative, warm, patient, understanding, dependable, trustworthy, professional, good at communicating and listening, hardworking, able to build rapport, empathetic, positive, and optimistic [99, 106, 109, 111, 117, 124, 130, 131, 133]. Negative attributes associated with negative experiences were service providers being patronizing in their approach, not genuine in their compassion or formulaic and generic in their approach, or having done an inadequate job discussing recovery [109, 121, 122]. Examples by innovation group are in Table 6.

#### Process

##### The importance of planning

The importance of planning was exemplified in examples of good and poor planning of the implementation process. Good planning was about anticipating known or expected challenges and building in processes for mitigating them [91, 95, 102, 103, 108, 109] Where implementation challenges were encountered these were associated with inadequate planning, particularly around the availability of protocols, guidelines, and/or clear information on roles, information management, and training [95, 112, 130, 134]. Examples by innovation group are in Table 7.

**Table 7:** Process: themes and examples from each innovation group

Innovation group	Theme^**a**^	Example
**Family-focused innovations**	The importance of planning	Early anticipation of issues with hiring new staff and effective planning (particularly the challenge of hiring staff for an innovation based on a model that did not yet exist in the community) helped to enhance workforce criteria over time [91].
	Early and continuous engagement with stakeholders	Engaging collaboratively with service providers to revise and refine the forms and protocols they would use as part of the innovation, helped ensure these were clear, simple, and adhered to [91].
**Peer workers**	The importance of planning	Planning was essential for mitigating known implementation challenges through well-chosen strategies such as having processes for embedding peer workers into the team (e.g. formal introductions, photos on walls) [109], anticipating staff concerns about peer workersʼ boundaries by discussing these in pre-implementation meetings and subsequent supervision [103], reducing role conflict and confusion with clear recruitment strategies [95, 102], policies on staff/client relationships, and operationalization of the peer role, and by providing training [102].
	Early and continuous engagement with stakeholders	Engaging with carer and clinician expert reference groups helped identify and select an intervention to be delivered by peer workers [109]. Peer workers felt they could have been engaged with more by being given a clearer leadership role in implementation to resolve issues of confusion, denial, and ineffective implementation of recovery practice [94].
**Personal recovery planning**	Early and continuous engagement with stakeholders	There was a need for greater, earlier, and more sustained engagement with funders, auditors, psychiatrists, admission and intake staff, and service users [109, 117, 119, 121, 124].
**Recovery colleges**	Early and continuous engagement with stakeholders	Engaging with organization staff early in the implementation process for their input into processes and procedures helped to leverage existing resources and prompt staff to offer classes in recovery colleges [127].
**Service navigation and coordination**	The importance of planning	Lack of adequate planning around protocols, work roles, information management, and training was an important contributor to the implementation problems encountered [130, 134].
	Early and continuous engagement with stakeholders	A lack of stakeholder engagement was highlighted as an implementation challenge. There was a need for greater engagement with stakeholders such as service users, families, and service providers in the planning stage to collaboratively develop elements such as protocols, work roles, responsibilities, required outcomes, information management, and service logistics and design [130, 134].

^a^If the studies in the innovation group did not contribute data to a theme, that theme is not listed under the innovation group and no example is provided

##### Early and continuous engagement with stakeholders

The importance of engaging with a variety of stakeholders early and continuously in the implementation process was mentioned in a number of studies across innovations [91, 109, 117, 119, 121, 124, 127, 130, 134]. Some studies highlighted successful engagement [91, 109, 127] whereas others noted that early and continuous engagement with stakeholders was lacking and needed [94, 109, 117, 119, 121, 124, 130, 134]. Successful engagement was around involving key stakeholders in selecting the innovation [91], refining associated materials [109], and leveraging resources [127]. Examples by innovation group are in Table 7.

## Discussion

Our review is, to the best of our knowledge, the first systematic review on the implementation of recovery-oriented services. Our synthesis has demonstrated how recovery has been operationalized into different innovations, and the common factors that influence its implementation. In terms of the characteristics of the recovery-oriented innovations, flexibility, relationship building, and lived experience are important factors to consider when designing innovations. At the level of organizations, traditional biomedical culture, staff turnover, available resources to support personal recovery goals, gaps in access to knowledge and information about new roles and procedures, and interpersonal relationships are essential factors to anticipate and plan for. The evidence to date also notes the issue of individual variation in recovery knowledge and the characteristics that make up recovery-oriented service providers. Finally, planning is key, as is engaging early with stakeholders and continuing to do so over the course of implementation. In this section. we will discuss some of these, and the CFIR constructs they relate to, in more detail, including how these findings compare to reviews of other interventions that used CFIR.

It is important to note that this was not a review of all the programs and services that exist in recovery, or the effectiveness of innovations, but rather was a review of research that has studied the process, experience, or factors that shape implementation. Some of the innovations identified are well established in some countries (peer workers) and some are new (e-innovations, recovery colleges). All help to operationalize recovery guidelines. Unlike innovations such as new treatments, the aim of recovery innovations is to transform mental health services towards a recovery orientation. By virtue of this, they are complex innovations primarily targeting deep culture change, not simply at the individual behavioural level but at the organization and system level. Many countries have committed in policy to implementing a recovery-orientation into their services [154] but knowing how to do this is the more challenging question. The literature synthesized here demonstrates how recovery as a policy, strategy, or approach has been operationalized into new recovery-specific innovations such as new training programs, new services, and new roles in the service system. Most aim to change wider organizational culture through these specific actions and many studies identified the importance of embedding implementation of these types of innovations within a wider commitment to recovery transformation [18, 94, 98, 99, 102, 107, 115, 119, 124, 135]. For transformation to happen, widespread change across organizations, from paperwork, to language, to hiring structures, need to change [155]. However, taking this on all at once is a daunting task, and decision-makers may prefer to make a start by introducing more tangible innovations like those included in this synthesis.

Like other systematic reviews, on different topics however, we used the CFIR as both a framework for extraction and synthesis [156–163]. Comparing our results to those of these reviews highlights important differences in what some constructs mean in the context of implementing recovery-oriented services compared to other interventions. For example, complexity in other reviews related to things like the length of consultations [156], difficult changes to workload, routines, and priorities [159], technical aspects like screening, resources, and number of professionals involved [157], and challenges with software and hardware [160]. In the case of recovery, complexity also related to managing relationships since a core characteristic of the innovations was making a change to the way service providers and service users interact. Similarly, culture was not reported on in depth in other reviews, whereas in our review it is highly significant across studies. This is likely because recovery is about system transformation and organizational culture change, and is quite a radical departure from traditional mental health services [48, 155]. Similar to other reviews using CFIR, included studies contributed the least data to outer setting [159, 161], and process [162].

Overall the CFIR, as a compilation of factors known to influence implementation, worked well as a data-extraction and synthesis framework, suggesting that implementation factors in the context of recovery-oriented services are similar to innovations in health and social care. However, in order to synthesize the evidence on implementing recovery into services we did have to adapt the CFIR framework- which itself is a contribution consistent with the best-fit framework synthesis method [72]. We replaced more medicalized terminology in the CFIR like “patients” and “intervention” with “service user” and “innovation”. Services should not intervene on someone’s recovery; they should support it through providing recovery-oriented services [49]. We also observed that service users (in CFIR terms “patients”) are inherently framed as outside the inner setting in CFIR—located explicitly in the outer setting in the 2009 version of CFIR we used [71]. The language of the CFIR definitions for the inner setting imply that organizational staff are the focus of the inner setting. However, from a recovery perspective, service users are actors within the inner setting and we took this view when extracting data. The result was that issues like service users perspectives’ on the learning climate were extracted to the inner setting and not to the outer setting or domain of individuals.

In terms of specific constructs, we added two. One was the sub-construct “engaging stakeholders” within the engaging construct in the process domain. While the “engaging” construct focuses on how stakeholders are attracted to participate in the innovation, the idea of engagement in the studies in this review related more closely to the recovery-oriented principle of co-production, that is engaging with stakeholders such as staff and service users to design and develop innovations [164, 165]. It is important to note that we used the 2009 version of CFIR [71], whereas more recent iterations have included the constructs “key stakeholders” and “patients and consumers” [166]. However, from a recovery perspective, we see no reason why consumers/service users should not inherently be considered key stakeholders. We also developed an additional construct within inner setting called “interpersonal relationships” out of data that did not fit elsewhere. Interpersonal relationships can refer to staff relationships (including with managers) or staff and service user relationships, goes beyond issues of communication and networks, and is an important part of the implementation climate. In the case of recovery, which aims to transform the way service users and service providers relate to one another [167, 168], the state of these relationships before and during implementation is an important implementation factor to consider.

### Review limitations

Because of the conceptual ambiguity surrounding recovery, and frequent misuse of the term [169], we had to keep our search criteria broad and found it conceptually challenging to determine when described programs were truly recovery-oriented and new, and when the word recovery was tokenistic or in fact referring to clinical recovery. Primary articles were inconsistent in providing a referenced definition of recovery-oriented services. We may have excluded studies that other reviewers would have included. Since we chose not to include systematic reviews or dissertations in this review, in retrospect we should not have searched the Cochrane Library or the ProQuest Dissertations and Theses databases.

Another conceptual challenge in this review was deciding on what constituted an implementation study in a field (mental health recovery) that has only recently begun intersecting with implementation science. Only 16 of the included studies used the word implementation in their title or keywords. This justifies why we did not rely on this label in our search to locate studies, but also signifies we are in an early stage of implementation research in this area. Another indication of this is the fact that we could only identify six studies that used an implementation-related framework in their research [103, 109, 112, 119, 134, 139]. By extension, the use of standard implementation terminology like that provided in the CFIR was infrequent. It was up to the reviewer extracting data to become intimately familiar with the CFIR construct definitions and see reference to them in the primary studies when the primary studies themselves were, for the most part, not using this terminology. If resources had been available, ideally two reviewers could have independently extracted data.

Another limitation may have been our decision to exclude pre-implementation studies, program descriptions, and grey literature. These may have included additional information on implementation factors, described other types of innovations, and have widened the geographical spread of studies. Finally, it is important to acknowledge that critical appraisal is a contested topic [170] but also a fundamental step in a systematic review [171]. Since critical appraisal is the result of two individuals’ judgements, our sensitivity analyses should be interpreted with due acknowledgement that scores could have been different had two other reviewers applied the MMAT.

### Recommendations for future research

The evidence base on the implementation of recovery into services to date has allowed us to identify important factors but not to study their exact mechanisms or effects, for example how exactly poor flexibility might lead to poor outcomes, or how greater flexibility may lead to better implementation outcomes. Research relating implementation factors to implementation outcomes is needed, as is research relating implementation barriers to implementation strategies. Stakeholders involved in implementation efforts can use tools like the CFIR-ERIC Matching Tool v.1 [166] to help prioritize strategies to consider including in their implementation plans. In this review, we screened program descriptions from regions like South America, and predict that within the coming years we will see additional research publications from non-English language countries evaluating the implementation of recovery. Future reviews and updates should pay particular attention to this emerging literature. Future research should also empirically study research participants’ perspectives on outer setting and process CFIR constructs. This synthesis found that we have the least evidence on these two domains. So far, primary study authors have tended to report outer setting and process issues factually as part of background or program description rather than explicitly targeting them in data collection. Lastly, mental health recovery researchers considering using the CFIR in their research may want to adopt some of the adaptations we describe in the discussion. These adaptations made the CFIR more compatible with mental health recovery in this review and may prove useful for future primary implementation research on recovery.

## Conclusions

This systematic mixed studies review has highlighted the factors known to influence the implementation of recovery-oriented services based on the evidence available to date. There are many types of innovations that operationalize recovery-transformation of services. This review identifies the factors that decision makers should consider in the domains of intervention characteristics, outer setting, inner setting, individuals, and process, regardless of the specific recovery-oriented innovation selected for implementation.

## Supplementary Information

**Additional file 1.** PRISMA Checklist and ENTREQ Checklist.

**Additional file 2.** Comparison of Protocol and Finished Review.

**Additional file 3.** Search Strategy MEDLINE.

**Additional file 4.** Modified CFIR Data Extraction Framework with Definitions.

**Additional file 5.** Reference List of Included Studies.

**Additional file 6.** Mixed Methods Appraisal Tool Results.

**Additional file 7.** Study Characteristics table.

**Additional file 8.** Illustrative Quotes for Each Theme.

## Data Availability

The datasets supporting the conclusions of this article are included within the article (and its additional files).

## Abbreviations

CFIR: Consolidated Framework for Implementation Research

## Footnotes

1 MW and ES appraised three of the 70 studies

2 Factors specific to individual types of innovations will be analysed and reported separately in future publications
