# Supplementary material for: Factors influencing the implementation of mental health recovery into services: a systematic mixed studies review
Source: Syst Rev. 2021 May 5;10:134. doi: 10.1186/s13643-021-01646-0 (PMC8101029; doi:10.1186/s13643-021-01646-0)
Supplement: Supplementary file 2 — Additional file 2. Comparison of Protocol and Finished Review. [file 13643_2021_1646_MOESM2_ESM.docx]

Additional file 3

Piat, M., Wainwright, M., Sofouli, E., Vachon, B., Deslauriers, T., Prefontaine, C., Frati, F. Factors influencing the implementation of mental health recovery into services: a systematic mixed studies review

**Key Points from Published Protocol and any Changes made in course of conducting the review**

|  | **PUBLISHED PROTOCOL** | **PUBLISHED REVIEW** |
| --- | --- | --- |
| AIM OF THE SYNTHESIS | how recovery is understood; challenges involved in implementation and, overall,  to what extent transformation to recovery oriented  services and systems is occurring. | Factors that influence the implementation of mental health recovery into services |
| SETTINGS and POPULATION | Inpatient, outpatient and community-based mental health settings. | No change |
|  | Studies will be included if they describe and evaluate the implementation of any intervention based on recovery principles that aims at transforming the orientation of  mental health services or organisations to a recovery approach. | Our focus is on new efforts to transform services. Long-standing programs that more recently incorporated recovery principles were excluded. |
|  | Studies on services for addiction populations will also be excluded, as recovery is conceptualised differently in the addictions field. | No change |
| EXTRACTION | Consolidated Framework for Implementation Science used at the conceptual framework | No change |
|  | Sample elements for data extraction appear below in table 1. The categories on the extraction grid include methodological  elements based on the PICO mnemonic (PICO=population, intervention, comparison, outcome).87  Also elements corresponding to the six research questions will be extracted and organised using the CFIR,79 a multilevel  five-dimension determinant framework88 that constitutes a highly useful tool for identifying barriers and facilitators influencing implementation outcomes. | No change  Data extracted only to the CFIR framework |
| OUTCOMES | Outcomes might include change in organisational culture; more integrated service networks and partnerships; increased knowledge, skills and/or attitudinal change among mental health providers; more use  of evidence-based recovery-oriented best practices; greater consumer/provider collaboration, consumer self-management  and evaluation. | Our review focuses on process rather than outcome and therefore synthesizes factors that shaped implementation, rather than effectiveness outcomes |
| LANGUAGE | No restriction | No change |
| SOFTWARE | Distiller for selection and extraction. NVivo for analysis | Distiller used for study selection. Descriptive extraction in Distiller, CFIR extraction in NVivo12 |
| HANDLING QUANT STUDIES | Thus, our overall approach will be to convert all the evidence into qualitative form.  The quantitative data will be transformed into qualitative form by extracting key concepts and findings within the elements geared to our research questions, as described above. | No change |
| ANALYSIS/SYNTHESIS | Analytic procedures and synthesis will follow a  three-stage process: (1) organisation of studies into logical categories according to their design, and methodology and coding using NVivo 11 software; (2) within-study analysis, according to the study questions; (3) cross-study synthesis of the data using an adaptation of the stepwise thematic analysis developed by Lucas *et al*,93 according to the following procedures: (1) two reviewers will independently review data collated under each of the research questions; (2) codes produced by each researcher will be compared and a consolidated list of themes produced for  each research question; (3) themes occurring under each question will be clustered around common dimensions; (4) results of the thematic analysis will be presented to  the research team at a consensus meeting.  Specific measures will be taken to enhance | Because of the decision to use CFIR for data extraction and synthesis we chose the Best Fit Framework Synthesis method which incorporates thematic analysis. The emphasis was not on answering pre-defined questions but on deductively coding data to the CFIR in the process of extraction followed by thematic analysis of data within each CFIR construct. The within-study analysis was replaced with a within-case analysis with the case being the innovation group. The cross-study synthesis was replaced by cross-case analysis, with cases being the synthesized findings for each innovation group. |
| TRUSTWORTHINESS, RELIABILITY, VALIDITY | Detailed descriptions, contextual material and the quality assessment of each paper will  also help readers make judgements about the reliability and validity of the data. | No change  In addition a sensitivity analysis was performed |
| QUALITY ASSESSMENT OF REVIEW | AMSTAR | Amstar was not used because it was created for RCT reviews.  Rather than use a quality assessment tool we applied reporting guidelines. Currently no reporting guideline exists for mixed-studies reviews so we drew on relevant items from ENTREQ and PRISMA. |
