# Supplementary material for: Factors influencing the implementation of mental health recovery into services: a systematic mixed studies review
Source: Syst Rev. 2021 May 5;10:134. doi: 10.1186/s13643-021-01646-0 (PMC8101029; doi:10.1186/s13643-021-01646-0)
Supplement: Supplementary file 3 — Additional file 3. Search Strategy MEDLINE. [file 13643_2021_1646_MOESM3_ESM.docx]

Additional file 4

Piat, M., Wainwright, M., Sofouli, E., Vachon, B., Deslauriers, T., Prefontaine, C., Frati, F. Factors influencing the implementation of mental health recovery into services: a systematic mixed studies review

**Search Strategy: MEDLINE**

Ovid MEDLINE(R) without Revisions and Epub Ahead of Print, In-Process & Other Non-Indexed Citations 1996 to Present

**Search name:** Recovery Services Final take 2_MEDLINE UPDATE

| 1. (recovery-oriented or recovery-orientated or recovery-orientation).ti,ab,kf. |  |
| --- | --- |
| 2. exp Mental Health Services/ |  |
| 3. recovery.ti,ab,kf. |  |
| 4. 2 and 3 |  |
| 5. mental disorders/ or adjustment disorders/ or exp anxiety disorders/ or exp dissociative disorders/ or exp mood disorders/ or exp neurotic disorders/ or exp personality disorders/ or exp "schizophrenia and disorders with psychotic features"/ or mental health/ or exp delirium/ or mentally ill persons/ |  |
| 6. (anxiety or depression or depressive or bipolar or schizophrenia or schizophrenic or phobia* or astheni* or delirium or schizoid or ((personality or mood or dissociative or neurotic or psychotic or panic or phobic or stress or adjustment or obsessive or compulsive) adj (disorder* or disease*))).ti,ab,kf. |  |
| 7. ((mental or psychiatric or psychologic*) adj (health or illness* or disorder* or disease* or problem or problems or issue or issues or well being)).ti,ab,kf. |  |
| 8. 5 or 6 or 7 |  |
| 9. health services/ or exp community health services/ |  |
| 10. "delivery of health care"/ or "delivery of health care, integrated"/ |  |
| 11. Health Services Administration/ or Program evaluation/ |  |
| 12. exp inservice training/ or patient care management/ or comprehensive health care/ or critical pathways/ or disease management/ or medication therapy management/ or patient care team/ |  |
| 13. (health* services or ((healthcare or health care) adj2 delivery)).ti,ab,kf. |  |
| 14. Self Care/ |  |
| 15. primary health care/ or patient-centered care/ |  |
| 16. Public Health/ |  |
| 17. (assertive community treatment* or Service level intentions).ti,ab,kf. |  |
| 18. ((Service* or organization* or organisation* or unit or units or department* or program* or clinic or clinics or ward or wards or staff or workforce or work force) adj2 (leader* or innovate* or innovation or objective* or change* or transforms or transform or transformation* or transformed or transforming or structure* or restructure* or structuring or restructuring or opportunit* or strength* or model or models or priority or priorities or policy or policies or procedure* or allocation or allocated or allocate or reform or reforms or needs or improve or improves or improved or improvement or assess* or responsive* or evaluat* or plan or plans or planning or planned or development or outcome*)).ti,ab,kf. |  |
| 19. (service* and (intervent* or assessment* or training or skill* or competenc*)).ti,ab,kf. |  |
| 20. (implementation or implementing or implemented or implement or implements).ti,ab,kf. |  |
| 21. (Recovery Self-Assessment or Recovery Enhancing Environment Measure or Recovery Oriented Systems Indicators or Recovery Interventions Questionnaire or Recovery Oriented Practices Index or Evaluation of the Collaborative Recovery Model or Recovery Promotion Fidelity Scale or Recovery Oriented Service Evaluation or Elements of a Recovery Facilitating System or Pillars of Recovery Service Audit Tool or Recovery Based Program Inventory or Recovery Culture Progress Report or Scottish Recovery Indicator or Staff Attitudes to Recovery Scale or Recovery Promoting Relationships Scale or Recovery Knowledge Inventory or Magelian Recovery Culture Report Card).ti,ab,kf. |  |
| 22. 9 or 10 or 11 or 12 or 13 or 14 or 15 or 16 or 17 or 18 or 19 or 20 |  |
| 23. 3 and 8 and 22 |  |
| 24. 1 or 4 or 21 or 23 |  |
| 25. limit 24 to yr="1998 -Current" |  |
| 26. (2016 12 2* or 2017* or 2018*).dt,ez. |  |
