# Supplementary material for: Factors influencing the implementation of mental health recovery into services: a systematic mixed studies review
Source: Syst Rev. 2021 May 5;10:134. doi: 10.1186/s13643-021-01646-0 (PMC8101029; doi:10.1186/s13643-021-01646-0)
Supplement: Supplementary file 4 — Additional file 4. Modified CFIR Data Extraction Framework with Definitions. [file 13643_2021_1646_MOESM4_ESM.docx]

**Additional file 5**

Piat, M., Wainwright, M., Sofouli, E., Vachon, B., Deslauriers, T., Prefontaine, C., Frati, F. Factors influencing the implementation of mental health recovery into services: a systematic mixed studies review

**Data-Extraction Framework and Definitions**

| **Consolidate Framework for Implementation Science (CFIR)**  **DOMAINS AND CONSTRUCTS**  **(Damschroder et al., 2009)** | **Adapted definitions for use as a data extraction framework** |
| --- | --- |
| **INTERVENTION CHARACTERISTICS** | |
| **Source of the intervention** | Authors’ or research participants’ descriptions/reports of the source of the intervention. |
| **Evidence strength and quality** | Authors’ or research participants’ descriptions/reports of the quality and validity of evidence supporting the belief that the intervention will have desired outcomes. |
| **Relative advantage** | Authors’ or research participants’ descriptions/reports of the advantage of implementing the intervention versus an alternative solution. |
| **Adaptability** | Authors’ or research participants’ descriptions/reports of the degree to which the intervention can be adapted, tailored, refined, or reinvented to meet local needs. |
| **Trialability** | Authors’ or research participants’ descriptions/reports of the ability to test the intervention on a small scale in the organization and to be able to reverse course (undo implementation) if warranted. |
| **Complexity** | Authors or research participants’ descriptions/reports of perceived difficulty of implementation, reflected by duration, scope, radicalness, disruptiveness, centrality, and intricacy and number of steps required to implement. |
| **Design quality and packaging** | Authors’ or research participants’ descriptions/reports of perceived excellence in how the intervention is bundled, presented and assembled. |
| **Cost** | Authors’ or research participants’ descriptions /reports of the costs associated with implementation, including the cost of both developing and implementing the intervention and the associated “opportunity costs” (e.g. costs/benefits of implementing this intervention vs. alternative interventions, options that may have been considered). |
| **Additional information** | Any further information about the characteristics of the intervention as described/reported by authors or research participants. |
| **OUTER SETTING** | |
| **Patient needs and resources** | Authors’ or research participants’ descriptions/reports of the extent to which service user needs, as well as barriers and facilitators to meet those needs, are accurately known and prioritized by the organization. (including patients’ own perceptions) |
| **Cosmopolitanism** | Authors’ or research participants’ descriptions/reports of the organization’s networks/links to other external organizations. |
| **Peer pressure** | Authors’ or research participants’ descriptions/reports of any mimetic or competitive pressure to implement the intervention (typically because most or other key peer or competing organizations have already implemented or are in a bid for a competitive edge.) |
| **External policies & incentives** | Authors’ or research participants’ descriptions/reports of any external strategies employed to spread the intervention (e.g. policy and regulations (governmental or other central entity), external mandates, recommendations and guidelines, pay-for-performance, collaboratives, and public or benchmark reporting) |
| **Additional information** | Any further information about the Outer Setting (e.g. other relevant features of the economic, political and social context) as described/reported by authors or research participants. |
| **INNER SETTING** | |
| **Structural characteristics** | Authors’ or research participants’ descriptions/reports of organizational structure (social architecture, age, maturity and size of an organization, team structure, staff turnover). |
| **Networks and communications** | Authors’ or research participants’ descriptions/reports of the nature and quality of webs of social networks and the nature and quality of formal and informal communications within an organization. |
| **Culture** | Authors’ or research participants’ descriptions/reports of the organizational norms, values, basic assumptions and general climate. |
| **Implementation climate** | Authors’ or research participants’ descriptions/reports of the absorptive capacity for change, shared receptivity of involved individuals to an intervention, and the extent to which use of that intervention will be rewarded, supported, and expected within their organization. |
| **Tension for change** | Authors’ or research participants’ descriptions/reports of stakeholders’ perception of the current situation as intolerable or needing change. |
| **Compatibility** | Authors’ or research participants’ descriptions/reports of the tangible fit between the meaning and values attached to the intervention by involved individuals, and individuals’ own norms, values, and perceived risks and needs, and how the intervention fits with existing workflows and systems. |
| **Relative priority** | Authors’ or research participants’ descriptions/reports of individuals’ shared perception of the importance of the implementation within the organization. (including individuals’ own perceptions) |
| **Organizational incentives and rewards** | Authors’ or research participants’ descriptions/reports of extrinsic incentives such as goal-sharing awards, performance reviews, promotions, and raises in salary, and less tangible incentives such as increased stature or respect. |
| **Goals and feedback** | Authors’ or research participants’ descriptions/reports of the degree to which goals were clearly communicated, acted upon, and fed back to staff, and alignment of that feedback with goals. |
| **Learning climate** | Authors’ or research participants’ descriptions/reports of the learning climate, for example the degree to which: leaders expressed their own fallibility and need for team members’ assistance and input; team members felt that they were essentially valued, and knowledgeable partners in the change process; individuals felt psychologically safe to try new methods; and the degree to which there was sufficient time and space for reflective thinking and evaluation. |
| **Readiness for implementation** | Authors’ and research participants’ descriptions/reports of tangible and immediate indicators of organizational commitment to its decision to implement an intervention. |
| **Leadership engagement** | Authors’ and research participants’ descriptions/reports of the commitment, involvement, and accountability of leaders and managers with the implementation. |
| **Available resources** | Authors’ and research participants’ descriptions/reports of the level of resources dedicated for implementation and on-going operations including money, training, education, physical space, and time. |
| **Access to knowledge and information** | Authors’ and research participants’ descriptions/reports of the ease of access to digestible information and knowledge about the intervention and how to incorporate it into work tasks. |
| **Additional information** | Any further information related to the Inner Setting described/reported by authors and research participants. |
| **CHARACTERISTICS OF INDIVIDUALS** | |
| **Knowledge and beliefs about the intervention** | Authors’ or research participants’ descriptions/reports of individuals’ attitudes toward the intervention, the value they placed on the intervention, and their familiarity with facts, truths and principles related to the intervention. (including individuals’ reports of their own attitudes) |
| **Self-efficacy** | Authors’ or research participants’ descriptions/reports of individuals’ belief in their own capabilities to execute courses of action to achieve implementation goals. (including individuals’ reports of their own beliefs) |
| **Individual stage of change** | Authors’ or research participants’ descriptions/reports of the phase an individual is in, as he or she progresses toward skilled, enthusiastic, and sustained use of the intervention. (including individuals’ own characterization of the phase they are in) |
| **Individual identification with organization** | Authors’ or research participants’ descriptions/reports of how individuals perceive the organization, their relationship with the organization and their degree of commitment to the organization. |
| **Other personal attributes** | Authors’ or participants’ descriptions/reports of individuals’ personal traits such as tolerance of ambiguity, intellectual ability, motivation, values, competence, capacity, and learning style. (including individuals’ own descriptions/reports of their personal traits) |
| **Additional information** | Any further information related to individuals as described/reported by authors or research participants |
| **PROCESS** | |
| **Planning** | Authors’ or research participants’ descriptions/reports of the degree to which a scheme or method of behavior and tasks for implementing an intervention are developed in advance, and the quality of those schemes or methods. |
| **Engaging** | Authors’ and research participants’ descriptions/reports of how appropriate individuals were attracted to, and involved in, implementation and use of the intervention (e.g. social marketing, education, role modeling, training, and other similar activities) |
| **Opinion leaders** | Authors’ and research participants’ descriptions/reports of the participation of opinion leaders in the intervention – that is, individuals in an organization who have formal or informal influence on the attitudes and beliefs of their colleagues with respect to implementing the intervention. (including opinion leaders’ own descriptions/reports of their participation) |
| **Formally appointed internal implementation leaders** | Authors’ and research participants’ descriptions/reports of the participation of formally appointed internal implementation leaders in the intervention – that is individuals formally appointed with responsibility for implementing an intervention as coordinator, project manager, team leader, or other similar role. (including formally appointed leaders’ own descriptions/reports of their participation) |
| **Champions** | Authors’ and research participants’ descriptions/reports of the participation of champions in the intervention– that is individuals who dedicate themselves to supporting, marketing, and driving through an implementation while overcoming indifference or resistance that the intervention may provoke in an organization. (including champions’ own descriptions/reports of their participation) |
| **External Change Agents** | Authors’ and research participants’ descriptions/reports of the participation of external change agents in the intervention– that is individuals who are affiliated with an outside entity who formally influence or facilitate intervention decisions in a desirable direction. (including external change agents’ own descriptions/reports of their participation) |
| **Engaging with stakeholders** | Authors’ and research participants’ descriptions/reports of engagement with diverse stakeholders (e.g. health professionals, family, patients/clients) to achieve stakeholder ‘buy-in’ for the intervention. |
| **Executing** | Authors’ and research participants’ descriptions/reports of the degree to which the implementation was carried-out or accomplished according to plan. |
| **Reflecting and evaluating** | Authors’ and research participants’ descriptions/reports of processes for collecting and feeding back about the progress and quality of implementation (e.g. regular personal and team debriefing about progress and experience) |
| **Additional information** | Any further information related to the implementation process as described/reported by authors or research participants. |
