# Supplementary material for: Factors influencing the implementation of mental health recovery into services: a systematic mixed studies review
Source: Syst Rev. 2021 May 5;10:134. doi: 10.1186/s13643-021-01646-0 (PMC8101029; doi:10.1186/s13643-021-01646-0)
Supplement: Supplementary file 5 — Additional file 5. Reference List of Included Studies. [file 13643_2021_1646_MOESM5_ESM.docx]

Additional file 6

Piat, M., Wainwright, M., Sofouli, E., Vachon, B., Deslauriers, T., Prefontaine, C., Frati, F. Factors influencing the implementation of mental health recovery into services: a systematic mixed studies review

**Reference List of Included Studies**

1. Ahern CC, Bieling P, McKinnon MC, McNeely HE, Langstaff K. A Recovery-Oriented Care Approach: Weighing the Pros and Cons of a Newly Built Mental Health Facility. Journal of psychosocial nursing and mental health services. 2016;54(2):39-48.

2. Ashman M, Halliday V, Cunnane JG. Qualitative Investigation of the Wellness Recovery Action Plan in a UK NHS Crisis Care Setting. Issues in Mental Health Nursing. 2017;38(7):570-7.

3. Banfield M, Forbes O. Health and social care coordination for severe and persistent mental illness in Australia: a mixed methods evaluation of experiences with the Partners in Recovery Program. International Journal of Mental Health Systems. 2018;12:13.

4. Beehler S, Marsella SA, Henderson PM, Resnick SG, Meterko M. Factors contributing to the effective functioning of veterans mental health councils. Psychological Services. 2018;10:10.

5. Biebel K, Nicholson J, Woolsey K, Wolf T. Shifting an agency’s paradigm: Creating the capacity to intervene with parents with mental illness. American Journal of Psychiatric Rehabilitation. 2016;19(4):315-38.

6. Bromage B, Kriegel L, Williamson B, Maclean K, Rowe M. Project Connect: A community intervention for individuals with mental illness. American Journal of Psychiatric Rehabilitation. 2017;20(3):218-33.

7. Byrne L, Happell B, Reid-Searl K. Recovery as a Lived Experience Discipline: A Grounded Theory Study. Issues in Mental Health Nursing. 2015;36(12):935-43.

8. Byrne L, Roennfeldt H, O'Shea P, Macdonald F. Taking a Gamble for High Rewards? Management Perspectives on the Value of Mental Health Peer Workers. International Journal of Environmental Research & Public Health [Electronic Resource]. 2018;15(4):13.

9. Cameron J, Hart A, Brooker S, Neale P, Reardon M. Collaboration in the design and delivery of a mental health Recovery College course: experiences of students and tutors. Journal of Mental Health. 2018:1-8.

10. Chapman SA, Blash LK, Mayer K, Spetz J. Emerging Roles for Peer Providers in Mental Health and Substance Use Disorders. American Journal of Preventive Medicine. 2018;54(6S3):S267-S74.

11. Chinman M, Salzer M, O'Brien-Mazza D. National survey on implementation of peer specialists in the VA: implications for training and facilitation. Psychiatric Rehabilitation Journal. 2012;35(6):470-3.

12. Choy-Brown M, Hamovitch EK, Cuervo C, Stanhope V. I Have My Own Lease--So Why the Service Plan Again? Perspectives on Service Planning in Supportive Housing. Psychiatric Rehabilitation Journal. 2016;39(4):313-20.

13. Cleary M, Raeburn T, Escott P, West S, Lopez V. 'Walking the tightrope': The role of peer support workers in facilitating consumers' participation in decision-making. International Journal of Mental Health Nursing. 2018;27(4):1266-72.

14. Clossey L, Gillen J, Frankel H, Hernandez J. The experience of certified peer specialists in mental health. Social Work in Mental Health. 2016;14(4):408-27.

15. Dalgarno M, Oates J. The meaning of co- production for clinicians: an exploratory case study of Practitioner Trainers in one Recovery College. Journal of Psychiatric & Mental Health Nursing. 2018;15:15.

16. Dunstan D, Anderson D. Applying Strengths Model principles to build a rural community-based mental health support service and achieve recovery outcomes. Rural & Remote Health. 2018;18(1):3708.

17. Eikmeier G, Kieser LD, Paap M, Utschakowski J, Lacroix A. Reorganising a department of psychiatry according to recovery principles: A pilot study with mixed-method design. Zeitschrift fur Evidenz Fortbildung und Qualitat im Gesundheitswesen. 2017;120:16-20.

18. Ellison ML, Glickman ME, Chinman M, Mitchell-Miland C, Schutt RK, Schultz MR, et al. Patterns and Predictors of Engagement in Peer Support Among Homeless Veterans With Mental Health Conditions and Substance Use Histories. Psychiatric Rehabilitation Journal. 2016;39(3):266-73.

19. Foster K, Isobel S. Towards relational recovery: Nurses' practices with consumers and families with dependent children in mental health inpatient units. International Journal of Mental Health Nursing. 2018;27(2):727-36.

20. Gammon D, Strand M, Eng LS, Børøsund E, Varsi C, Ruland C. Shifting Practices Toward Recovery-Oriented Care Through an E-Recovery Portal in Community Mental Health Care: A Mixed-Methods Exploratory Study. Journal of Medical Internet Research. 2017;19(5):1-.

21. Gates LB, Akabas SH. Developing strategies to integrate peer providers into the staff of mental health agencies. Adm Policy Ment Health. 2007;34(3):293-306.

22. Gilburt H, Slade M, Bird V, Oduola S, Craig TK. Promoting recovery-oriented practice in mental health services: a quasi-experimental mixed-methods study. BMC Psychiatry. 2013;13:167.

23. Hamilton AB, Chinman M, Cohen AN, Oberman RS, Young AS. Implementation of consumer providers into mental health intensive case management teams. J Behav Health Serv Res. 2015;42(1):100-8.

24. Hamilton S, Tew J, Szymczynska P, Clewett N, Manthorpe J, Larsen J, et al. Power, choice and control: How do personal budgets affect the experiences of people with mental health problems and their relationships with social workers and other practitioners? British Journal of Social Work. 2016;46(3):719-36.

25. Hungerford C, Fox C. Consumer's perceptions of Recovery-oriented mental health services: an Australian case-study analysis. Nurs Health Sci. 2014;16(2):209-15.

26. Hungerford C, Hungerford A, Fox C, Cleary M. Recovery, non-profit organisations and mental health services: 'Hit and miss' or 'dump and run'? International Journal of Social Psychiatry. 2016:8.

27. Hungerford C, Kench P. The perceptions of health professionals of the implementation of Recovery-oriented health services: a case study analysis. Journal of Mental Health Training, Education & Practice. 2013;8(4):208-18.

28. Hungerford C, Richardson F. Operationalising recovery-oriented services: The challenges for carers. Advances in Mental Health. 2013;12(1):11-21.

29. Hurley E, McKay EA. Research article: The recognition and adoption of the recovery approach by occupational therapists in acute psychiatric settings in Ireland. Irish Journal of Occupational Therapy. 2009;37(2):5-13.

30. Hurley J, Cashin A, Mills J, Hutchinson M, Kozlowski D, Graham I. Qualitative study of peer workers within the 'Partners in Recovery' programme in regional Australia. International Journal of Mental Health Nursing. 2018;27(1):187-95.

31. Isaacs AN, Sutton K, Dalziel K, Maybery D. Outcomes of a care coordinated service model for persons with severe and persistent mental illness: A qualitative study. International Journal of Social Psychiatry. 2017;63(1):40-7.

32. Khoury E, Rodriguez del Barrio L. Recovery-Oriented Mental Health Practice: A Social Work Perspective. British Journal of Social Work. 2015:i127-i44.

33. Kidd SA, McKenzie K, Collins A, Clark C, Costa L, Mihalakakos G, et al. Advancing the recovery orientation of hospital care through staff engagement with former clients of inpatient units. Psychiatr Serv. 2014;65(2):221-5.

34. Kido Y, Kayama M. Consumer providers' experiences of recovery and concerns as members of a psychiatric multidisciplinary outreach team: A qualitative descriptive study from the Japan Outreach Model Project 2011-2014. PLoS ONE [Electronic Resource]. 2017;12(3):e0173330.

35. Kisely S, Wyder M, Dietrich J, Robinson G, Siskind D, Crompton D. Motivational aftercare planning to better care: Applying the principles of advanced directives and motivational interviewing to discharge planning for people with mental illness. International Journal of Mental Health Nursing. 2017;26(1):41-8.

36. Korsbek L, Tonder ES. Momentum: A smartphone application to support shared decision making for people using mental health services. Psychiatric Rehabilitation Journal. 2016;39(2):167-72.

37. Lamont E, Harris J, McDonald G, Kerin T, Dickens GL. Qualitative investigation of the role of collaborative football and walking football groups in mental health recovery. Mental Health and Physical Activity. 2017;12:116-23.

38. Lawn S, Smith A, Hunter K. Mental health peer support for hospital avoidance and early discharge: An Australian example of consumer driven and operated service. Journal of Mental Health. 2008;17(5):498-508.

39. Le Boutillier C, Slade M, Lawrence V, Bird VJ, Chandler R, Farkas M, et al. Competing priorities: staff perspectives on supporting recovery. Administration and policy in mental health. 2015;42(4):429-38.

40. Leamy M, Clarke E, Le Boutillier C, Bird V, Janosik M, Sabas K, et al. Implementing a complex intervention to support personal recovery: a qualitative study nested within a cluster randomised controlled trial. PLoS ONE [Electronic Resource]. 2014;9(5):e97091.

41. Lodge AC, Kaufman L, Stevens Manser S. Barriers to Implementing Person-Centered Recovery Planning in Public Mental Health Organizations in Texas: Results from Nine Focus Groups. Adm Policy Ment Health. 2016:1.

42. Mahler L, Jarchov-Jadi I, Gervink A, Ayilmaz H, Wilfer A, Sischka K, et al. Multiperspectivity and peers on acute wards. [German]. Nervenheilkunde. 2015;34(4):249-52.

43. Mak WWS, Chan RCH, Pang IHY, Chung NYL, Yau SSW, Tang JPS. Effectiveness of Wellness Recovery Action Planning (WRAP) for Chinese in Hong Kong. American Journal of Psychiatric Rehabilitation. 2016;19(3):235-51.

44. Mancini MA. An Exploration of Factors that Effect the Implementation of Peer Support Services in Community Mental Health Settings. Community Mental Health Journal. 2018;54(2):127-37.

45. Mandiberg JM, Gates LB. A community of practice for peer mental workers: Lessons learned. American Journal of Psychiatric Rehabilitation. 2017;20(2):156-74.

46. Marshall SL, Oades LG, Growe TP. Australian mental health consumers contributions to the evaluation and improvement of recovery-oriented service provision. Isr J Psychiatry Relat Sci. 2010;47(3):198-205.

47. McFarland L, Fenton A. Unfogging the future: Investigating a strengths-based program to build capacity and resilience in parents with mental illness. Advances in Mental Health. 2018:No Pagination Specified.

48. McKenna B, Furness T, Dhital D, Park M, Connally F. The transformation from custodial to recovery-oriented care: a paradigm shift that needed to happen. J. 2014;10(4):226-33.

49. Milton A, Lloyd-Evans B, Fullarton K, Morant N, Paterson B, Hindle D, et al. Development of a peer-supported, self-management intervention for people following mental health crisis. BMC Research Notes. 2017;10(1):588.

50. Peer JE, Gardner M, Autrey S, Calmes C, Goldberg RW. Feasibility of implementing a recovery education center in a Veterans Affairs medical center. Psychiatric Rehabilitation Journal. 2018;41(2):135-40.

51. Perkins AM, Ridler JH, Hammond L, Davies S, Hackmann C. Impacts of attending recovery colleges on NHS staff. Mental Health & Social Inclusion. 2017;21(1):18-24.

52. Piat M, Lal S. Service providers' experiences and perspectives on recovery-oriented mental health system reform. Psychiatric Rehabilitation Journal. 2012;35(4):289-96.

53. Reed TA, Broussard B, Moore A, Smith KJ, Compton MT. Community navigation to reduce institutional recidivism and promote recovery: initial evaluation of opening doors to recovery in Southeast Georgia. Psychiatr Q. 2014;85(1):25-33.

54. Reid R, Escott P, Isobel S. Collaboration as a process and an outcome: Consumer experiences of collaborating with nurses in care planning in an acute inpatient mental health unit. International Journal of Mental Health Nursing. 2018;27(4):1204-11.

55. Salkeld R, Wagstaff C, Tew J. Toward a new way of relating: an evaluation of recovery training delivered jointly to service users and staff. Journal of Mental Health. 2013;22(2):165-73.

56. Scanlan JN, Hancock N, Honey A. Evaluation of a peer-delivered, transitional and post-discharge support program following psychiatric hospitalisation. BMC Psychiatry. 2017;17(1):307.

57. Siantz E, Henwood B, Gilmer T. Implementation of peer providers in integrated mental health and primary care settings. Journal of the Society for Social Work and Research. 2016;7(2):231-46.

58. Siantz E, Henwood B, Gilmer T. Peer Support in Full-Service Partnerships: A Multiple Case Study Analysis. Community Mental Health Journal. 2017;53(5):542-9.

59. Simpson A, Oster C, Muir-Cochrane E. Liminality in the occupational identity of mental health peer support workers: A qualitative study. International Journal of Mental Health Nursing. 2018;27(2):662-71.

60. Smith-Merry J, Freeman R, Sturdy S. Implementing recovery: an analysis of the key technologies in Scotland. International Journal of Mental Health Systems. 2011;5(1):11.

61. Stewart V, Slattery M, Roennfeldt H, Wheeler AJ. Partners in Recovery: paving the way for the National Disability Insurance Scheme. Australian Journal of Primary Health. 2018;06:06.

62. Strand M, Gammon D, Eng LS, Ruland C. Exploring Working Relationships in Mental Health Care via an E-Recovery Portal: Qualitative Study on the Experiences of Service Users and Health Providers. JMIR Mental Health. 2017;4(4):e54.

63. Thomas N, Farhall J, Foley F, Leitan ND, Villagonzalo KA, Ladd E, et al. Promoting Personal Recovery in People with Persisting Psychotic Disorders: Development and Pilot Study of a Novel Digital Intervention. Frontiers in psychiatry Frontiers Research Foundation. 2016;7:196.

64. Uppal S, Oades LG, Crowe TP, Deane FP. Barriers to transfer of collaborative recovery training into Australian mental health services: implications for the development of evidence-based services. J Eval Clin Pract. 2010;16(3):451-5.

65. Wallace G, Bird V, Leamy M, Bacon F, Boutillier C, Janosik M, et al. Service user experiences of REFOCUS: a process evaluation of a pro-recovery complex intervention. Social Psychiatry & Psychiatric Epidemiology. 2016;51(9):1275-84.

66. Willging CE, Lamphere L, Rylko-Bauer B. The transformation of behavioral healthcare in New Mexico. Adm Policy Ment Health. 2015;42(3):343-55.

67. Williams A, Fossey E, Farhall J, Foley F, Thomas N. Recovery After Psychosis: Qualitative Study of Service User Experiences of Lived Experience Videos on a Recovery-Oriented Website. JMIR Mental Health. 2018;5(2):e37.

68. Williams V, Deane FP, Oades LG, Crowe TP, Ciarrochi J, Andresen R. A cluster-randomised controlled trial of values-based training to promote autonomously held recovery values in mental health workers. Implement Sci. 2016;11:13.

69. Young AS, Chinman M, Forquer SL, Knight EL, Vogel H, Miller A, et al. Use of a consumer-led intervention to improve provider competencies. Psychiatr Serv. 2005;56(8):967-75.

70. Zabel E, Donegan G, Lawrence K, French P. Exploring the impact of the recovery academy: a qualitative study of Recovery College experiences. Journal of Mental Health Training, Education & Practice. 2016;11(3):162-71.
