# Supplementary material for: Factors influencing the implementation of mental health recovery into services: a systematic mixed studies review
Source: Syst Rev. 2021 May 5;10:134. doi: 10.1186/s13643-021-01646-0 (PMC8101029; doi:10.1186/s13643-021-01646-0)
Supplement: Supplementary file 7 — Additional file 7. Study Characteristics table. [file 13643_2021_1646_MOESM7_ESM.docx]

Additional file 8

Piat, M., Wainwright, M., Sofouli, E., Vachon, B., Deslauriers, T., Prefontaine, C., Frati, F. Factors influencing the implementation of mental health recovery into services: a systematic mixed studies review

**Study Characteristics Table**

| **Author(s)/Title of Included Studies** | **Innovation group^[[1]](#footnote-1)^** | **Country** | **Study Setting** | **Innovation** | **Study Design (for MMAT^[[2]](#footnote-2)^)** | **Data sources and sample** |
| --- | --- | --- | --- | --- | --- | --- |
| Ahern et al. (2016). A Recovery-Oriented Care Approach: Weighing the Pros and Cons of a Newly Built Mental Health Facility. | Architecture | Canada | Tertiary-care, inpatient mental health hospital, integrated with outpatient mental health, diagnostic, and medical services | Newly built facility designed to be patient centered | Mixed-Methods | Focus groups:  - 39 patients and family members  Surveys:  - 101 patients and visitors  Key informant interviews:  - 14 directors and managers  Naturalistic observations  - 72 hours  Hospital records: pass use |
| Ashman et al. (2017). Qualitative Investigation of the Wellness Recovery Action Plan in a UK NHS Crisis Care Setting. | Personal Recovery Planning | UK | NHS Mental health crisis resolution and home treatment teams (CRHTs) | The Wellness Recovery Action Plan (WRAP) was introduced into a CRHT to improve resilience-building, foster learning from crisis and promote recovery-oriented working. | Qualitative | Semi-structured interviews:  - 6 service users who had undertaken the CRHT course of WRAP education. |
| Banfield & Forbes. (2018). Health and social care coordination for severe and persistent mental illness in Australia: a mixed methods evaluation of experiences with the Partners in Recovery Program. | Service navigation and coordination | Australia | Consortium of local organisations and service providers within local regions, coordinated by a “Lead Agency” such as a primary health network | Partners in Recovery Program. Wrap-around care to people with severe and persistent mental illness and complex care needs. | Mixed Methods | Quantitative validated questionnaires:  - 25 clients  - 14 service providers  Semi-structured interviews:  - 6 clients  - 2 carers  - 4 service providers |
| Beehler et al. (2018). Factors contributing to the effective functioning of veterans mental health councils. | Consumer-led advisory council | USA | Veterans Affairs Medical Centres | Veterans Mental Health Council as mechanism for veteran  families and community stakeholders to provide input into the  structure and operations of local VA mental health services | Qualitative | Phone interviews:  - 7 council members  - 8 VA-employed staff liaisons |
| Biebel et al. (2016). Shifting an agency’s paradigm: Creating the capacity to intervene with parents with mental illness. | Family-focused innovation | USA | Community mental health agency | Family Options - a family-centered care management intervention - 24-hour a day, 7-days-a-week support and advocacy through home visits and a telephone warm line, and a small pool of discretionary funding to be used flexibly. | Qualitative | Ethnographic interviews:  - 2 agency administrators (total of 48 interviews)  - 2 program directors (total of 9 interviews)  - 3 family coaches (total of 29 interviews)  - 1 administrative assistant (total of 4 interviews)  - 1 clinical consultant (total of 10 interviews) |
| Bromage et al. (2017). Project Connect: A community intervention for individuals with mental illness | Community connections | USA | Local mental health authority | Project Connect: support for people with mental illness in  creating and sustaining connections in their communities | Qualitative | Semi-structured interviews and demographic questionnaires:  - 30 participants in Project Connect |
| Byrne et al. (2015). Recovery as a Lived Experience Discipline: A Grounded Theory Study. | Peer workers | Australia | Lived experience-run organisations, non-government organisations, and government organisations. | Employment of people with lived experience in mental health sector | Qualitative | In-depth interviews:  -13 individuals employed to work from a lived experience perspective. |
| Byrne et al. (2018). Taking a Gamble for High Rewards? Management Perspectives on the Value of Mental Health Peer Workers. | Peer workers | Australia | Non profits and public health organizations | Systemic advocacy positions, education and training, and peer support workers (including some peer-run services) | Qualitative | In-depth face-to-face interviews:  - 25 people in traditional and designated peer executive or senior management roles.  1 focus group:  - 6 individuals employed in both peer designated and non-peer designated management roles |
| Cameron et al. (2018). Collaboration in the design and delivery of a mental health Recovery College course: experiences of students and tutors. | Recovery colleges | UK | National Health Service | Recovery college course | Qualitative | Semi-structured interviews:  - 9 students  - 4 tutors  Naturally occurring data:  - 30 pieces (flip chart notes and homework documentation) |
| Chapman et al. (2018). Emerging Roles for Peer Providers in Mental Health and Substance Use Disorders. | Peer workers | USA | 29 clinical (e.g. crisis stabilization units, psychiatric hospital) and non-clinical (e.g. peer-run respites, community centers) settings. | Peers in typical job duties including leading wellness groups, teaching classes, case management, and one on-one services | Qualitative | Interviews:  -194 state policymakers, directors of training and certification bodies, peer providers, and other staff in mental health and substance use treatment and recovery organizations.  Document review |
| Chinman et al. (2012). National survey on implementation of peer specialists in the VA: implications for training and facilitation. | Peer workers | USA | Veteran's administration system services | Employment of Peer Specialists assigned to clinical teams | Quantitative/ Descriptive | Survey:  - 92 out of 238 Local Recovery Coordinators (completion rate of 67%) |
| Choy-Brown et al. (2016). I Have My Own Lease--So Why the Service Plan Again? Perspectives on Service Planning in Supportive Housing. | Personal recovery planning | USA | 4 permanent supportive housing programs | Person centered care planning implemented as a replacement of standard care and service planning. | Qualitative | Focus groups: - 38 tenants (in 4 groups)  - 8 service coordinators (in 2 groups)  - 4 supervisors (in 1 group)  - 7 leaders (in 1 group) |
| Cleary et al. (2018). 'Walking the tightrope': The role of peer support workers in facilitating consumers' participation in decision-making. | Peer workers | Australia | Psychiatric hospitals and/or community mental health systems | Peer support workers in adult mental health services | Qualitative | Semi-structured interviews:  – 6 peer support workers |
| Clossey et al. (2016). The experience of certified peer specialists in mental health. | Peer workers | USA | Unclear. “mental health organizations” | Certified peer specialists (CPS) are mental health consumers who work as paid mental health staff to support other consumers | Qualitative | Three focus groups:  - 10 peer support workers  Individual interviews:  - 3 peer support workers |
| Dalgarno & Oates. (2018). The meaning of co- production for clinicians: an exploratory case study of Practitioner Trainers in one Recovery College. | Recovery colleges | UK | National Health Service | Recovery College | Qualitative | Semi-structured interviews:  - 8 clinicians who have co-produced and co-delivered workshops with a Recovery College Peer Trainer. |
| Dunstan & Anderson. (2018). Applying Strengths Model principles to build a rural community-based mental health support service and achieve recovery outcomes. | Service navigation and coordination | Australia | Funded by the Australian Government Department of Families, Housing, Community Services and Indigenous Affairs (FaHCSIA) for delivery by non-government organisations | Personal Helpers and Mentors (PHaMs) service - a non-clinical, community-based initiative. PHaMs caseworkers support and mentor people 'at risk of falling through the service gaps | Mixed Methods | Documents:  - 9 documents for staff orientation or collaboration.  Anonymized service data:  - 126 service users  Semi-structured interviews (with rating questions):  - 17 service users |
| Eikmeier et al. (2017). Reorganising a department of psychiatry according to recovery principles: A pilot study with mixed-method design. | Peer workers | Germany | General hospital, Psychiatric department, inpatient and outpatient clinics | "Recovery companions" -peer support workers were integrated in existing mental health teams | Qualitative (also has quantitative component but data not extracted as not relevant to review) | Interviews:  - 13 pre and 15 post with  stakeholders of the change management |
| Ellison et al. (2016). Patterns and Predictors of Engagement in Peer Support Among Homeless Veterans With Mental Health Conditions and Substance Use Histories. | Peer workers | USA | Department of Housing and Urban Development—Veterans Health Administration Supportive Housing Program (HUD/VASH) program | Veteran peer specialists provided 1 hr weekly one-on-one unstructured and structured meetings with veterans in the housing for up to 9 months. | Quantitative / Descriptive | Demographic data:  - 50 veterans in supported housing program  - Contact data recorded by each peer worker |
| Foster & Isobel. (2018). Towards relational recovery: Nurses' practices with consumers and families with dependent children in mental health inpatient units. | Family-focused innovations | Australia | Four mental health inpatient units in one local health district in New South Wales, comprising three acute adult inpatient units and one rehabilitation setting. | Family rooms - deﬁned spaces, often ﬁtted out with child-friendly furniture, toys, and resources, for children and families to use while visiting parent consumers in inpatient settings | Qualitative | Semi-structured in-depth interviews:  - 20 nurses |
| Gammon et al. (2017). Shifting Practices Toward Recovery-Oriented Care Through an E-Recovery Portal in Community Mental Health Care: A Mixed-Methods Exploratory Study. | E-innovations | Norway | Primary health care and specialist levels of care | ReConnect E-recovery portal. The portal consists of a toolbox,  anonymous peer support discussion forum, and messaging with  providers. | Mixed Methods | Online Questionnaire (demographics, use of internet and multiple psychosocial measures):  - 29 service users  Administrative data:  - user log  - forum postings  Focus group interviews:  - 27 service users and service providers (3 focus groups with each respondent group)  Documents:  - minutes of meetings and personal communications |
| Gates & Akabas. (2007). Developing strategies to integrate peer providers into the staff of mental health agencies. | Peer workers | USA | 27 Community-mental health agencies | Employment of peer providers in mental health agencies | Qualitative | Semi-structured telephone interviews:  - 93 staff were interviewed (27 executive directors, 18 HR representatives, 22 supervisors and 26 line staff).  Focus groups:  - 15 peer workers (in 2 groups) |
| Gilburt et al. (2013). Promoting recovery-oriented practice in mental health services: a quasi-experimental mixed-methods study. | Staff training | UK | Twenty two community- based and in-patient rehabilitation adult mental health teams for the inner-city London Boroughs of Lambeth. | Four full-day workshops in a classroom setting followed by an in-team half day session. Trainers with both professional expertise and lived experience. | Mixed Methods | Quantitative care plan audit:  - 673 care plans  Semi-structured interviews:  - 16 participating staff members |
| Hamilton et al. (2015). Implementation of consumer providers into mental health intensive case management teams. | Peer workers | USA | Veteran Health Administration, Six Mental Health Intensive Case Management teams attached to one Veterans Integrated Service Network | PEER Project (Peers Enhancing Recovery). Employment of consumer providers to do case management | Qualitative | Individual interviews:  - 8 service providers  - 5 five consumer providers  - 2 clients  - 4 consumer providers part of research team  Focus groups:  - 2 with clients |
| Hamilton et al. (2016). Power, choice and control: How do personal budgets affect the experiences of people with mental health problems and their relationships with social workers and other practitioners? | Personal budgets | UK | Three English local authorities | Personal  budgets for people with mental health problems | Qualitative | In-depth interviews:  - 52 service users  - 28 mental health practitioners |
| Hungerford et al. (2016). Recovery, non-profit organisations and mental health services: 'Hit and miss' or 'dump and run'? | Perspectives on implementing recovery-oriented services in general | Australia | Community-based Organization | Recommendations for implementing recovery-oriented services in accordance with strategic direction set by the national government (Australian Health Ministers, 2009) and action plan for involvement of consumers and carers | Qualitative | Focus groups:  - 15 community workers (in 4 groups) |
| Hungerford & Fox. (2014) Consumer's perceptions of Recovery-oriented mental health services: an Australian case-study analysis. | Perspectives on implementing recovery-oriented services in general | Australia | Public mental health service with inpatient units and community-based services | Recommendations for implementing recovery-oriented services in accordance with strategic direction set by the national government (Australian Health Ministers, 2009) and action plan for involvement of consumers and carers | Qualitative | Focus groups:  6 mental health consumers  Interviews:  - 3 mental health consumers |
| Hungerford & Kench. (2013). The perceptions of health professionals of the implementation of Recovery-oriented health services: a case study analysis. | Perspectives on implementing recovery-oriented services in general | Australia | Mental health service organisation across bed-based and community settings | Recommendations for implementing recovery-oriented services in accordance with strategic direction set by the national government (Australian Health Ministers, 2009) and action plan for involvement of consumers and carers | Qualitative | Focus Groups:  - [unclear number, but max 13]: nurses, occupational therapists, psychologists, social workers, managers. (in 4 groups)  Interview:  - 1 staff member |
| Hungerford & Richardson. (2013). Operationalising recovery-oriented services: The challenges for carers. | Perspectives on implementing recovery-oriented services in general | Australia | Four inpatient facilities and a range of community mental health services | Recommendations for implementing recovery-oriented services in accordance with strategic direction set by the national government (Australian Health Ministers, 2009) and action plan for involvement of consumers and carers | Qualitative | Semi-structured interviews:  - 10 carers of people with mental health problems. |
| Hurley & McKay. (2009). Research article: The recognition and adoption of the recovery approach by occupational therapists in acute psychiatric settings in Ireland. | Perspectives on implementing recovery-oriented services in general | Ireland | Acute psychiatric settings | The 2008 framework document ‘A Recovery Approach with the Irish Mental Health services’ divided service development into six key pillars (MHC, 2008). | Qualitative | Semi-structured interviews:  - 8 Occupational Therapists  - Field notes from interviews |
| Hurley et al. (2018). Qualitative study of peer workers within the 'Partners in Recovery' programme in regional Australia. | Service navigation and coordination  &  Peer workers | Australia | National mental health programme currently being linked into the National Disability Insurance Scheme. PIR nominates one organization as the practice lead, which is then responsible for supervision, consultation, quality assurance. | The Partners In recovery (PIR) program employs workers who have lived experience and Support Facilitators (SF) work at the system level to improve coordination and integration in support of consumer recovery. | Qualitative | Interviews:  - 4 peer workers  - 4 consumers  - 4 managers  - 10 support facilitators |
| Isaacs et al. (2017). Outcomes of a care coordinated service model for persons with severe and persistent mental illness: A qualitative study. | Service navigation and coordination | Australia | Gippsland Primary Health Network (GPHN), a regional health planning organization and the lead agency for PIR initiative in Gippsland, formed a regional PIR Consortium with the Community Mental Health Support Services (CMHSS) and the Area Mental Health Service. | Partners in Recovery (RIP) aimed to support a better integrated mental health care system by improving referral pathways, strengthening partnerships between the different services that would work together and promote a community-based recovery model. | Qualitative | Interviews:  - 3 coordinators of the initiative from Gippsland Primary Health Network  -15 support facilitators  - 2 support facilitators’ managers  - 12 other support providers (3 housing workers, 4 case managers, 1 drug & alcohol worker, 1 family services, 1 private psychologist, 2 personal helpers and mentors),  - 7 clients  - 6 carers |
| Khoury & Rodriguez del Barrio. (2015). Recovery-Oriented Mental Health Practice: A Social Work Perspective. | Perspectives on implementing recovery-oriented services in general | Canada | General Practice Clinic/Primary Care | Quebec Mental Health Action Plan (MHAP). | Qualitative | Semi-structured interviews:  -11 mental health social work practitioners (7 social workers, 3 managers, 1 clinical coordinator)  - 2 key informants  Document analysis  - internal mission statements, team structure, practice descriptions, government documents |
| Kidd et al. (2014). Advancing the recovery orientation of hospital care through staff engagement with former clients of inpatient units. | Staff training | Canada | Psychiatric Hospital Schizophrenia program. This service provides extensive outpatient services and includes six inpatient units | Series of talks (N=58) to in-patient staff by 12 former patients on a diverse range of recovery-related experiences and feed-back on experiences they had in the units | Mixed Methods | Self-assessment questionnaires for staff:  - before: Intervention group n= 37; Control group n=22)  - after (Intervention group n=22;Control group n=38)  Individual interviews:  - [number not clear] speakers  Focus groups:  - 20 staff |
| Kido & Kayama. (2017). Consumer providers' experiences of recovery and concerns as members of a psychiatric multidisciplinary outreach team: A qualitative descriptive study from the Japan Outreach Model Project 2011-2014. | Peer workers | Japan | Psychiatric multidisciplinary outreach teams | Consumer providers join psychiatric multidisciplinary teams aimed at reducing hospitalization or re-hospitalization. | Qualitative | Semi-structured interviews:  - 9 consumer providers |
| Kisely et al. (2017). Motivational aftercare planning to better care: Applying the principles of advanced directives and motivational interviewing to discharge planning for people with mental illness. | Personal recovery planning | Australia | 3 inpatient wards (1 intervention ward, 2 control wards) | Advanced care planning and Motivational Interviewing were combined into a single intervention Motivational Aftercare Planning (MAP) and applied them to discharge planning. | Qualitative | Audit of anonymized consecutive recovery plans retrieved from the Queensland Health’s electronic health record: - 100 intervention ward plans  - 197 control ward plans  Interviews:  - 20 service users |
| Korsbek & Tonder. (2016). Momentum: A smartphone application to support shared decision making for people using mental health services. | E-innovation | Denmark | Public Mental Health Services: a long-term rehabilitation ward, a treatment center for young people with psychosis or psychosis-like symptoms, a mental health community center | Momentum: smartphone app for shared decision-making | Qualitative | Focus groups:  - 12 multidisciplinary staff members (in 3 groups) - 7 doctors (in 1 group)  Individual interviews:  - 7 consumers |
| Lamont et al. (2017). Qualitative investigation of the role of collaborative football and walking football groups in mental health recovery. | Sports | UK | National Health Service boards in Scotland. Football groups hosted in community centres, local community sports centre, and inpatient psychiatric settings | Collaborative  mental health football (soccer) projects. Staff and service users collaboratively organize group and play together. | Qualitative | Focus groups:  - 18 service users  - 7 staff members |
| Lawn et al. (2008). Mental health peer support for hospital avoidance and early discharge: An Australian example of consumer driven and operated service. | Peer workers | Australia | Three general public hospitals supported by a community-based emergency team and three multidisciplinary community mental health teams | Peer Service for hospital avoidance and early discharge. | Quantitative and Qualitative | Administrative data:  - bed days saved  - crisis service contact  - emergency department presentations  - readmission rates.  Phone questionnaires and focus groups. [numbers for each group not clearly reported]  - consumers  - six carers  - peer support workers  - mental health staff  - eight GPs  - mentored peer coordinator  - Metro Home Link staff |
| Le Boutillier et al. (2015). Competing priorities: staff perspectives on supporting recovery. | Perspectives on implementing recovery-oriented services in general | UK | National Health Service | Sample is from REFOCUS trial. | Qualitative | Focus groups:  - 34 multidisciplinary clinicians and 21 team leaders (in 10 groups)  Interviews  - 18 clinicians  - 6 team leaders  - 8 senior managers |
| Leamy et al. (2014). Implementing a complex intervention to support personal recovery: a qualitative study nested within a cluster randomised controlled trial. | Staff training | UK | 14 community-based mental health teams in two National Health Service sites (one urban, one semi-rural) who received the intervention. | REFOCUS trial- Component 1: Recovery-promoting relationships, Component 2: Working practices | Qualitative | Interviews:  - 28 staff and team leaders from implementation teams  - 3 trainers  Focus Groups:  - 24 members of 4 implementation teams (in 4 groups)  Trainers’ reports:  - 14 Personal Recovery and 14 Coaching for Recovery Training reports |
| Lodge et al. (2016). Barriers to Implementing Person-Centered Recovery Planning in Public Mental Health Organizations in Texas: Results from Nine Focus Groups. | Personal recovery planning | USA | One state hospital and two community mental health centres | Person-centered recovery planning (PCRP) ‘‘a collaborative process between the person and his or her supporters (including the clinical practitioner) that results in the development and implementation of an action plan to assist the person in achieving his or her unique, personal goals along the journey of recovery’’ | Qualitative | Focus groups:  - 71 staff members (leaderships, practitioners and peer specialists) (in 9 groups) |
| Mahler et al. (2015). Multiperspectivity and peers on acute wards. [German]. | Peer workers | Germany | General hospital, inpatient psychiatric units | Recovery oriented Weddinger Model. Peers were a component of multi-profes­sional primary-care teams on acute wards. | Quantitative Descriptive | Questionnaires:  - 25 peer workers and service providers |
| Mak et al. (2016). Effectiveness of Wellness Recovery Action Planning (WRAP) for Chinese in Hong Kong. | Personal recovery planning | Hong Kong | NGO - New Life Psychiatric Rehabilitation Association (NLPRA). | Wellness Recovery Action Planning (eight 1.5-hour sessions) | Qualitative (the study was predominantly quantitative but quant findings not extracted as not related to our review) | Focus groups:  - 6 staff members (in 2 groups) |
| Mancini (2018). An Exploration of Factors that Effect the Implementation of Peer Support Services in Community Mental Health Settings. | Peer workers | USA | Agencies providing community based mental health services | Integration of peer services into community mental health settings | Qualitative | In-depth semi-structured interviews:  - 23 certified peer specialists  - 11 non-peer community mental health workers |
| Mandiberg & Gates. (2017). A community of practice for peer mental workers: Lessons learned. | E-innovation | USA | Not applicable | E-community of practice for peer workers | Qualitative | Administrative data:  - 14 users’ aggregated use data and website contributions  - Website moderator notes |
| Marshall et al. (2010). Australian mental health consumers contributions to the evaluation and improvement of recovery-oriented service provision. | Personal recovery planning | Australia | four government and five  non-government organizations within New South Wales,  Queensland and Victoria, Australia. | The Collaborative Recovery Model (CRM) and associated training program, including training to use the tool Collaborative Goal Technology (CGT) | Qualitative | Focus groups:  - 18 mental health consumers |
| McFarland & Fenton. (2018). Unfogging the future: Investigating a strengths-based program to build capacity and resilience in parents with mental illness. | Family-focused innovation | Australia | Community health organization | Two co-facilitators delivered five, two-hour long, weekly modules, in a face to face sessions aimed at building capacity and resilience in parents with mental illness. | Qualitative | Weekly reflective activities and a demographic survey:  - 4 parents  Telephone interviews:  - 2 program facilitators |
| McKenna et al. (2014). The transformation from custodial to recovery-oriented care: a paradigm shift that needed to happen. | Perspectives on implementing recovery-oriented services in general | Australia | Secure inpatient service within a large metropolitan mental health organization | The domains of recovery were superimposed onto the continuum of care providing a manualized “how to” guide for each phase of the continuum | Qualitative | Interviews:  - 4 consumers  - 1 consumer consultant  - 1 unit manager (a mental health nurse)  - 1 lead nurse  - 3 registered nurses  - 1 enrolled nurse  - 1 consultant psychiatrist  - 1 social worker  - 1 occupational therapist  - 1 psychologist  - 1 service quality improvement manager  Written records and documents:  - minutes of meetings, surveys, project briefs, project reports, unit policies, memos, and service-wide policies or protocols. |
| Milton et al. (2017). Development of a peer-supported, self-management intervention for people following mental health crisis. | Personal recovery planning  &  Peer workers | UK | Crisis resolution teams within 10 National Health Service Trusts | A peer supported, self-management intervention for people leaving Crisis Resolution team services. | Qualitative | Interviews:  - 41 CRT service users (stage 2)  - 9 CRT service users (stage 4)  - 18 CRT services users (stage 5)  Focus groups:  - [number of participants unclear] service users, staff and carers (stage 3) [in 12 groups]  - 4 peer workers (repeated at each stage |
| Peer et al. (2018). Feasibility of implementing a recovery education center in a Veterans Affairs medical center. | Recovery colleges | USA | National Service, VA Medical Centre | Recovery education centre | Quantitative Descriptive | Administrative data:  - 781 veterans referred to Recovery College Services (referral, enrollment, engagement, utilization, rate of no show, tabulating add/drop).  Focus groups:  - veterans (number not known) (in 2 groups) |
| Perkins et al. (2017). Impacts of attending recovery colleges on NHS staff. | Recovery colleges | UK | National Heath Service | Recovery College | Quantitative Descriptive | Online survey:  - 94 National Health Service staff who had enrolled onto a course |
| Piat & Lal. (2012). Service providers' experiences and perspectives on recovery-oriented mental health system reform. | Perspectives on implementing recovery-oriented services in general | Canada | Inpatient, outpatient, and community-based settings | Range of interventions in each site: strategic plan, official recovery program within its clinical services, recovery “model” for its case management services, recovery as guiding principles, strengths-based model | Qualitative | Focus groups and socio-demographic questionnaires :  - 68 service providers (in 9 groups) |
| Reed et al. (2014). Community navigation to reduce institutional recidivism and promote recovery: initial evaluation of opening doors to recovery in Southeast Georgia. | Service navigation and coordination  &  Peer workers | USA | Three community service boards/agencies serving a 34-county region | Opening Doors to Recovery (ODR) program aims to prevent recidivism of consumers in and out of hospitals, the criminal justice system, and homelessness, while promoting recovery. | Qualitative | In-depth Interviews:  - 23 key stakeholders (including four consumers, six navigators and four referring mental health professionals) |
| Reid et al. (2018). Collaboration as a process and an outcome: Consumer experiences of collaborating with nurses in care planning in an acute inpatient mental health unit. | Personal recovery planning | Australia | 25-bed, mixed-gender, acute adult inpatient unit | Collaborative care planning- a process that involves nurses and consumers working together to identify individual goals and strategies for recovery. | Qualitative | Semi-structured interviews:  - 12 service users |
| Salkeld et al. (2013). Toward a new way of relating: an evaluation of recovery training delivered jointly to service users and staff. | Staff training | UK | Public community-based mental health services within one National Health Service trust | A training program designed by trainers with lived experienced and delivered jointly to staff and service users. It comprised three 1-day workshops. | Qualitative | In-depth interviews:  - 4 healthcare professional participants who had participated in the training. |
| Scanlan et al. (2017). Evaluation of a peer-delivered, transitional and post-discharge support program following psychiatric hospitalisation. | Peer workers | Australia | Non-government mental health service | Hospital to Home (H2H). Staff from inpatient teams referred individuals to H2H who needed additional support. Delivered by a people with lived experience. | Qualitative (quant data related to effectiveness outcomes and not extracted for this review) | Telephone semi-structured interviews:  - 17 service users |
| Siantz et al. (2016). Implementation of peer providers in integrated mental health and primary care settings. | Peer workers | USA | Community mental health centers | 24 integrated pilot programs that included peer workers on their staff. | Qualitative | Qualitative program reports from full-day site visits which included:  - semi-structured interviews (not voice recorded)  - focus groups (no detail on the number of focus groups or attendees)  - observation  - chart reviews  - program document review  - field notes |
| Siantz et al. (2017). Peer Support in Full-Service Partnerships: A Multiple Case Study Analysis. | Peer workers | USA | Full Service Partnerships, team-based mental health service models. | Peers working in Full Service Partnerships (FSPs). FSPs combined supported housing with team-based treatment model and the expectation do “whatever it takes” to promote recovery | Qualitative | In-depth semi-structured interviews:  - 8 peer providers  - 8 program directors |
| Simpson et al. (2018). Liminality in the occupational identity of mental health peer support workers: A qualitative study. | Peer workers | UK | National Health Service, four psychiatric wards | Peer Support Workers providing face-to-face and telephone support during the transition period from inpatient to community care for 4 weeks | Qualitative | Two focus groups (at month 3 and 7):  - 8 peer support workers  Semi-structured interviews:  - peer support coordinator (interviewed twice)  - 13 service users |
| Smith-Merry et al. (2011). Implementing recovery: an analysis of the key technologies in Scotland. | Perspectives on implementing recovery-oriented services in general  &  Peer workers  &  Personal recovery planning | UK | National Health Service | Peer Support, WRAP, Scottish Recovery Indicator, Recovery Narratives | Qualitative | Semi-structured interviews:  - 9 practitioners, government policy makers, individuals working within non-governmental advocacy organisations and service users/community activists.  Documentary sources:  - policy documents, reviews and working papers on recovery |
| Stewart et al. (2018). Partners in Recovery: paving the way for the National Disability Insurance Scheme. | Service navigation and coordination | Australia | 10 Partners in Recovery organizations in the Brisbane South Primary Health Network (BSPHN) | Partners In Recovery program. Support facilitators (SF) employed to coordinate care and assist participants to identify their needs, and develop a person-centred action plan, locate and coordinate access to the services required and be a point of contact for the PIR participant and their families | Qualitative | Semi-structured interviews:  -14 participants/clients in PIR  - 17 members of the participant’s support network  - 3 members of a consumer and carer advisory group |
| Strand et al. (2017). Exploring Working Relationships in Mental Health Care via an E-Recovery Portal: Qualitative Study on the Experiences of Service Users and Health Providers. | E-innovation | Norway | Primary care and specialist-level care | ReConnect E-recovery portal. The portal consists of a toolbox,  anonymous peer support discussion forum, and messaging with  providers. | Qualitative | Total of 31 participants (14 service users + 17 health providers)  Focus groups (6 groups):  - 11 service users  - 14 service providers  Individual interviews:  - 11 service users  - 8 service providers  Dyad interviews:  - 1 service provider and 1 service user together |
| Thomas et al. (2016). Promoting Personal Recovery in People with Persisting Psychotic Disorders: Development and Pilot Study of a Novel Digital Intervention. | E-innovation | Australia | Unclear | SMART (Self-Management and Recovery Technology) website). Website to be used on a tablet computer by mental health  workers to structure therapeutic discussions about personal recovery. | Quantitative / Descriptive | Administrative data:  - 10 users who completed all sessions’ proportion of sessions in which the website was used, assessed by the site’s record of log-on records during times appointments were held.  - site’s record of log-ons between appointments.  - Rate of and reasons for dropout from the intervention  Multiple validated questionnaires (see article)  Phone interview (unclear) |
| Uppal et al. (2008). Barriers to transfer of collaborative recovery training into Australian mental health services: implications for the development of evidence-based services. | Personal recovery planning | Australia | Four community-based governmental and six non-governmental mental health services | Collaborative Recovery Training Program (CRTP). A core aspect of the CRTP was training clinicians in the use of the two protocols: Collaborative Goal Technology (CGT) and Home- work (HW). | Quantitative / non-RCT | Open-ended survey and quantitative questionnaires:  - 173 clinicians  .  Administrative data:  - clinical audit of protocol implementation |
| Wallace et al. (2016). Service user experiences of REFOCUS: a process evaluation of a pro-recovery complex intervention. | Personal Recovery Planning | UK | Twenty-seven community mental health teams | REFOCUS. Manualised, pro-recovery intervention, delivered to whole community mental health teams. Two components: pro-recovery Working Practices and Recovery-promoting relationships. The Working Practices (WPs). Working Practices included Supporting goal-striving (WP3). | Qualitative | Interviews:  - 24 service users  Focus groups:  - 13 service users (in 2 groups) |
| Willging et al. (2015). The transformation of behavioral healthcare in New Mexico. | Service navigation and coordination | USA | Public-sector behavioral healthcare in New Mexico. | Comprehensive community support services (CCSS), clinical homes (CHs), and core service agencies (CSAs) initiatives were intended to facilitate a wraparound approach to planning services | Qualitative | Study 1: Document analysis of key texts concerning the three initiatives –  Narrative Interviews:  - 25 state officials  - 325 personnel  -15 executive staff of ValueOptions  - 10 CSA-specific participants (6 provider agency administrators, 2 state officials, and 2 from OptumHealth New Mexico   Study 2: Focus groups:  - 22 clinical homes providers  - 25 juvenile justice professionals  Semi-structure interviews:  - 9 clinical homes providers  - 3 coaches  - 5 juvenile justice judges |
| Williams et al. (2016). Recovery After Psychosis: Qualitative Study of Service User Experiences of Lived Experience Videos on a Recovery-Oriented Website. | Staff training | Australia | Four community-managed organisations that provide programmes to support individuals with severe and recurrent mental health challenges | Collaborative Recovery Model training is an evidence-based staff development programme structured around six core principles or workplace values. Participants assigned to the values group received a third day of training that comprised a structured values clarification card sorting task | Quantitative / RCT | Likert-scale questionnaires to measure autonomous motivation and plans to implement:  - 146 Mental health workers |
| Williams et al. (2018). A cluster-randomised controlled trial of values-based training to promote autonomously held recovery values in mental health workers. | E-innovation | Australia | Community-based Organization | SMART (Self-Management and Recovery Technology) website | Qualitative | Semi structured interviews:  - 36 service users (6 of 36 interviewed twice) |
| Young et al. (2005). Use of a consumer-led intervention to improve provider competencies. | Staff training | USA | 5 large community mental health organizations in two states, each with a number of sites. Project sites were part of provider organizations that provided care by using Medicaid funds managed by ValueOptions, a large behavioral health care organization. | Staff Supporting Skills for SelfHelp, a consumer-led intervention which was designed to improve provider quality, empower mental health consumers, and promote mutual support. The intervention included education, clinician-client dialogues,ongoing technical assistance, and support of self-help. | Qualitative (included quant component but outcomes not extracted for this review as not related to review question) | Pre and post semi-structured interviews:  - 16 staff (upper managers, middle managers, direct supervisors,and front-line clinicians). |
| Zabel et al. (2016). Exploring the impact of the recovery academy: a qualitative study of Recovery College experiences. | Recovery colleges | UK | National Health Service Trust | Recovery Academy | Qualitative | Focus groups:  - 14 Staff member/health professionals (in 2 groups)  - 10 persons with lived experience (including one family member) (in 2 groups) |

1. These were the groups assigned by reviewers as part of the synthesis process. Innovations were “cases”. Some studies were categorized to more than one case. [↑](#footnote-ref-1)
2. The study design category indicated reflects the version of the mixed-methods appraisal tool (MMAT) we used. Studies that used both quantitative and qualitative data collection methods are categorized here as “Qualitative” if the quantitative component did not relate to our review question (e.g. effectiveness outcomes). [↑](#footnote-ref-2)
