# Supplementary material for: Factors influencing the implementation of mental health recovery into services: a systematic mixed studies review
Source: Syst Rev. 2021 May 5;10:134. doi: 10.1186/s13643-021-01646-0 (PMC8101029; doi:10.1186/s13643-021-01646-0)
Supplement: Supplementary file 8 — Additional file 8. Illustrative Quotes for Each Theme. [file 13643_2021_1646_MOESM8_ESM.docx]

Additional file 10

Piat, M., Wainwright, M., Sofouli, E., Vachon, B., Deslauriers, T., Prefontaine, C., Frati, F. Factors influencing the implementation of mental health recovery into services: a systematic mixed studies review

**Illustrative Quotes for Each Theme**

| **CIFR Domain and Theme** | **Illustrative Quotes from Contributing Studies** |
| --- | --- |
| INTERVENTION CHARACTERISTICS | |
| **Flexibility** | I don’t suppose anyone’s going to feel exactly the same every week. Sometimes you might really like to say, I don’t want to talk about how I feel, can we just look at the plan? Or, I just want to talk about how I feel, I don’t want to look at the plan, that kind of thing. To be flexible I think is probably the most important, but a bit of both. (SU [service user] 28). (Milton et al. 2017, p.7) |
| **Relationship-building** | Through the process of socializing with someone  who has had similar experiences, the PSW [peer support worker]–service user relationship often developed into one resembling friendship:  It wasn’t.. .professional, and I think that’s what they appreciate the most, just a friend.  (PSW [peer support worker]) It’s like having a mate, a friend, you know? (Service user) The participants generally viewed this type of relationship positively. However, there could also be negative effects. Developing ‘friendships’ with peers was particularly problematic with the ending of the relationship, with some service users wanting to continue the ‘friendship’ and feeling ‘very sad, very, very sad’ (service user) and ‘gutted’ (service user) once the relationship ended. Similarly, the friend-like relationship led some PSW to feel as though they had mistreated their peers, as they too felt loss at the ending of the relationship: ‘I felt like I’d dumped somebody’ (PSW [peer support worker]). (Simpson et al. 2018, p.666) |
| **Lived-experience** | Participants appreciated the personal narrative from the facilitator who had lived experience of mental health difficulties. Witnessing their journey of recovery was thought to inspire hope and optimism in others: […] just the fact that there were people who were up there in the first session erm, co-facilitating who, who co-written the course, who’d, they were up there telling their story made me think that’s possible (P2). (Zabel et al. 2016, p. 167) |
| INNER SETTING | |
| **Traditional biomedical vs. recovery-oriented approach** | All nurses spoke of their role in addressing risk and protecting the safety of consumer parents, other consumers in the unit, and child and family visitors. They viewed this as an essential part of their role and accountability. Risks to family visitors were seen to include physical aggression and emotional distress from observed consumer behaviours, such as volatile, irritable, verbally abusive, and manic behaviours. Nurses were particularly concerned with child safety and risk of harm, with some identifying that colleagues actively discouraged child visits:  “I know for a fact that a lot of staff discourage visitation....A lot of what we do is risk management...the highest risk group would be the most vulnerable, so if you can stop them attending the unit, it’s one problem solved.”  (Nurse 20) (Foster & Isobel, 2018, p. 729) |
| **The importance of organizational and policy commitment to recovery-transformation** | Another respondent warned that a problem with current practices relating to WRAP [wellness recovery action planning] was that it was being instituted “in a traditional way in a traditional environment” (NGO 1). If WRAP was implemented within services that lacked a wider recovery  orientation, another respondent feared, it would become “just another care plan” (NGO 2). (Smith-Merry et al. 2011, p. 7) |
| **Staff turnover** | Leadership, peer, and practitioner participants at all three organizations also described several barriers related to the difﬁculty of implementing and sustaining cultural and organizational change. These concerns seemed to be particularly salient for leadership and peer specialist participants as well as participants from the state hospital. For example, a leadership participant from the state hospital noted:  “It’s just like the reality of having a sustained push in a public institution for several years. You know we had our particular challenges that I think we worked through wonderfully but I think that is a huge barrier. I mean if you look at how do you keep retention? And, you know, being able to have a continued vision for that amount of time. I think all of our challenges become a barrier to being able to do that.” (Lodge et al. 2016, p. 11) |
| **Lack of resources to support personal recovery goals** | However, some service providers thought the model was not without its challenges, including a lack of clarity of the precise role of the Support Facilitator and a lack of services available for coordination.  I think first you have to be sure that there are all those services to coordinate in the first place…Do all the services exist? … If you want to coordinate something there has to be something there to coordinate, I guess… For the model to work it has to be available. Service provider 4 (Banfield & Forbes, 2018, p. 9) |
| **Information gaps about new roles and procedures** | The experience of having little initial guidance on what was expected in the role of PW [peer worker]; including a job proﬁle and clariﬁcation of the type and volume of expected activity was ubiquitous across the PW participants. This was particularly evident in the initial 18 months of the PIR [Partners in Recovery] programme where the programme itself, the PIR staffs and those external agencies they interfaced with lacked clarity toward aspirations and mechanisms of PIR. PW (1) summed up the experience in the following statement related to the role: “I’ve basically had to develop it myself”. This was the case for those employed at the beginning, and those joining the programme within the ﬁrst 18 months. (Hurley et al. 2018, p. 189) |
| **Interpersonal relationships** | Some service users expressed frustration that providers repeatedly told them how busy they were as an excuse for why they had not worked with them through ReConnect. For example, in a café discussion:  “Why did [provider] agree to work with me through this tool if she never expected to do it? She should have just said no. You get so disappointed. That’s why it’s good to have each other [forum participants]—to call you my helpers. So we can share things.” (Gammon et al. 2017, p. 10)  Although some dyads clarified their understandings about how to use the portal and adjusted accordingly, others did not. Dyads that were enriched by use of ReConnect, despite not explicitly agreeing on how to use the system, appeared to have relationships that were open and adaptable at the outset. For some of those who experienced frustrations, the portal appeared to expose and sometimes reinforce suboptimal working relationships. (Strand et al., 2017, p. 10) |
| CHARACTERISTICS OF INDIVIDUALS | |
| **Variability in knowledge about recovery** | Alongside this, some of the interviewees raised concerns that many service users may not be “at the level” (Interviewee 4) where they can engage in such a training process – either because they are not sufficiently far forward in their own recovery journeys or because recovery, in the sense of no longer needing services, might not be a realistic goal for them. While this may indeed be a potential barrier from a service user perspective, it may also be that these perceptions are, at least in part, born out of apprehension and fears of staff, experiencing recovery concepts in practice themselves for the first time. (Salkeld et al. 2013, p. 169) |
| **Characteristics of recovery-oriented service providers** | PSWs [peer support workers] were valued by participants both for the warm relationship per se, and for the practical information and support they provided.  “And while everyone sort of was telling me that they would help, but no one really did anything, while she actually did things. And I really, really appreciate this” (PP01)  One participant was disappointed by his PSW’s inability to help with a housing problem, while two identified a difficult relationship per se with their PSW as a problem:  “I don’t like people telling me what… I’ll do it this week, I’ll do it that week. And you know, I’ve always been very independent and stuff. And I just find it a bit patronizing. So I’d rather, you know, do it my own, kind of, time, and frame…” (MP17) (Milton et al., 2017, p.13) |
| PROCESS | |
| **The importance of planning** | Agency administrators and the clinical consultant expressed concern about the challenges of hiring staff for an innovative intervention based on a model that, at that time, did not exist in the community. To address this challenge, they identified skill set criteria for the family coaches and the program director that included experience in the mental health and child welfare arenas, experience working with adults and children, and experience delivering strength-based, family-focused services. (Biebel et al. 2016, p. 325) |
| **Early and continuous engagement with stakeholders** | In the beginning, top administrators at the CH sites were  very enthusiastic about their participation, believing they would ﬁnd themselves ‘‘ahead of the curve’’ once the initiative was rolled out on a larger scale. Yet, they quickly became frustrated with the failure of state ofﬁcials and VONM [ValueOptions New Mexico] to fully articulate, up front, the roles, responsibilities, and outcomes required of their agencies. The majority observed in interviews and focus groups that their input regarding these expectations and outcomes had not been solicited. Once the CHs [clinical homes] were implemented, this lack of clarity created confusion for them and their agencies’ direct service providers, including the former case managers who  were now CCSS [comprehensive community support services] workers. As one clinical director conﬁded, ‘‘I am not sure any of us still know what it means to operate effectively as a CH.’’ Providers also criticized the state and VONM for creating rules ‘‘on the ﬂy’’ and not systematically disseminating them to all pilot test sites. (Willging et al., 2015, p. 350) |
